# Plastid and cytoplasmic origins of ^1^O_2_-mediated transcriptomic responses

**DOI:** 10.3389/fpls.2022.982610

**Published:** 2022-11-07

**Authors:** Eugene Koh, Alexander Brandis, Robert Fluhr

**Affiliations:** ^1^ Plant and Environmental Sciences, Weizmann Institute of Science, Rehovot, Israel; ^2^ Life Sciences Core Facility, Weizmann Institute of Science, Rehovot, Israel

**Keywords:** arabidopsis, singlet oxygen (1 O2), RNA oxidation, 8oxo-guanosine (8-oxoG), ROS - reactive oxygen species

## Abstract

The reactive oxygen species singlet oxygen, ^1^O_2_, has an extremely short half-life, yet is intimately involved with stress signalling in the cell. We previously showed that the effects of ^1^O_2_ on the transcriptome are highly correlated with 80S ribosomal arrest due to oxidation of guanosine residues in mRNA. Here, we show that dysregulation of chlorophyll biosynthesis in the *flu* mutant or through feeding by δ-aminolevulinic acid can lead to accumulation of photoactive chlorophyll intermediates in the cytoplasm, which generates ^1^O_2_ upon exposure to light and causes the oxidation of RNA, eliciting ^1^O_2_-responsive genes. In contrast, transcriptomes derived from DCMU treatment, or the *Ch1* mutant under moderate light conditions display commonalties with each other but do not induce ^1^O_2_ gene signatures. Comparing ^1^O_2_ related transcriptomes to an index transcriptome induced by cycloheximide inhibition enables distinction between ^1^O_2_ of cytosolic or of plastid origin. These comparisons provide biological insight to cases of mutants or environmental conditions that produce ^1^O_2_.

## Introduction

Reactive oxygen species (ROS) were previously thought to be by-products of cellular dysfunction, and their release exacerbated damage to cellular components, hastening cellular demise. In recent years, ROS have also been found to be produced in a regulated fashion and participate in multifarious cell signalling pathways. H_2_O_2_ is the most stable ROS and is regarded for its ability to take part in redox reactions [reviewed in (Foyer, 2018)]. The superoxide ROS radical ( ${\text{O}}_{2}^{-}$ ) has been shown to be produced in significant quantities in both chloroplasts and mitochondria, as a result of electron transport in photosynthesis and respiration, respectively. Additionally, it is produced by NADPH oxidases in various organelle membranes in response to pathogenic attack or during development [reviewed in (Kadota et al., 2015)], Such activity can trigger an organism-wide ‘ROS wave’ reminiscent of systemic signalling in the nervous system in animals (Miller et al., 2009). In contrast, singlet oxygen (^1^O_2_) has an extremely short half-life of 4 µs (Redmond & Kochevar, 2006), and was thought to be restricted to the action of light-driven photosensitization reactions typically occurring in the chloroplast (Hideg et al., 1998; Flors et al., 2006). Photosynthetically produced ^1^O_2_ was shown to damage photosynthetic machinery. In particular, the D1/2 reaction centre proteins appear to be particularly sensitive to ^1^O_2_ damage, and its *de novo* synthesis in response to damage is a critical repair mechanism to maintain chloroplast function [reviewed in (Li et al., 2018)]. Intriguingly, ^1^O_2_ was recently also shown to be produced in dark reactions (Mor et al., 2014; Prasad et al., 2017) and intimately related to osmotic stress responses in the root due to the activity of lipoxygenases (Chen and Fluhr, 2018; Chen et al., 2021).

^1^O_2_ signalling has been studied in a variety of systems, ranging from exogenous photosensitisers like Rose Bengal (RB) and Acridine Orange (AO), to genetic mutants such as FLUORESCENT IN BLUE LIGHT (*flu)*, CHLORINA 1 (*Ch1*) and to inhibitors of photosynthesis such as 3-(3,4-dichlorophenyl)-1,1-dimethylurea (DCMU). As ^1^O_2_ is an extremely short-lived molecule, and is highly reactive towards electron-rich biomolecules such as unsaturated fatty acids, the effects of the different treatments are restricted to the locality where they are generated. A recent study using chemooptogenetic methods to target photosensitisers to specific subcellular compartments highlighted the differential apoptotic and necrotic outcomes of ^1^O_2_ localization (Liang et al., 2020). In Arabidopsis, we previously showed that RB and AO localizes ^1^O_2_ production to the plasma membrane and vacuole respectively. AO, but not RB, resulted in the stimulation of vacuolar-driven cell death *via* the leakage of vacuolar proteases (Koh et al., 2016). Similarly, DCMU is an inhibitor of photosynthesis which blocks the electron transport chain of Photosystem II, preventing the conversion of light energy to ATP. Light energy remains trapped in the excited chlorophyll pigments, which then transfer this energy to nearby molecular oxygen, forming ^1^O_2_. This ^1^O_2_ can cause damage to the nearby photosynthetic apparatus, and prolonged exposure to light can ultimately lead to chlorophyll bleaching and cell death (Ridley, 1977).

The *flu* mutant was one of the earliest mutants used to characterise the effects of ^1^O_2_ production in the cell. This mutant bears a defect in the feedback regulation of δ-aminolevulinic acid (ALA) synthesis, which allows for uncontrolled protochlorophyllide (Pchd) accumulation in the dark (Meskauskiene et al., 2001). Normally in the dark, Pchd is sequestered in the chloroplast complexed with protochlorophyllide oxidoreductase proteins responsible for the safe phototransformation of Pchd to chlorophyllide (Masuda and Takamiya, 2004). However, excess unbound Pchd can act as a natural photosensitizer upon transition from dark to light. It transfers light energy to O_2_ *via* Type II reactions, producing copious amounts of ^1^O_2_ that promote cell death (op den Camp et al., 2003). Thus, the release of ^1^O_2_ is proportional to the amount of Pchd accumulation, as well as the intensity of light exposure (Wang and Apel, 2018). It was shown that a set of ^1^O_2_ sensitive genes were upregulated in response to dark/light transition in the *flu* mutant, which was distinct from that produced by another sources of ROS e.g. methyl viologen (MV), an inducer of superoxide formation (op den Camp et al., 2003). It was hypothesized that the response required cellular signal transduction as further second site mutants were discovered (executer mutants; *ex1, ex2*) which exhibited attenuation of cellular death and diminished stress gene expression (Wagner et al., 2004; Lee et al., 2007). It was also shown recently that oxidative modification of a specific tryptophan residue in EX1 was responsible for propagating the cell death response (Dogra et al., 2019). Similarly, the protease FtSH2 and grana localized protein SAFEGUARD1 (SAFE1) was also shown to play critical roles in regulating the *flu/ex1* response (Wang et al., 2016; Wang et al., 2020). The components of this regulatory pathway (Pchd, *flu*, *ex1/ex2*, FtSH2, SAFE1) have all been shown to reside within the confines of the chloroplast. Thus, it was proposed that some yet uncharacterised component was responsible for eliciting a nuclear-generated transcriptome response and provide for retrograde signalling.

Another source of ^1^O_2_ that occurs during photosynthesis was investigated in the *Ch1* mutant that lacks chlorophyll a oxygenase, resulting in plants deficient in chlorophyll b (Espineda et al., 1999). As a consequence, it is devoid of Photosystem II (PSII) chlorophyll-protein antenna complexes (Havaux et al., 2007). This results in the improper assembly of the normal architecture of the PSII system which renders the mutant extremely sensitive to photooxidative stress (Havaux et al., 2007). The exposure of the mutant to a combination of HL/cold stress for 2 days resulted in production of ^1^O_2_, coupled with an increase in measured lipid peroxidation and stress gene transcript levels. The sensitivity of this mutant to photooxidative stress can be exacerbated by reduction of cellular ROS scavengers, which was demonstrated by crossing the *Ch1* mutants with scavenger deficient mutants such as *vte2* (tocopherol) and *pdx1.2* (vitamin B6) (Havaux et al., 2007; Havaux et al., 2009). Interestingly, the crossing of *Ch1* with the *ex1* mutant did not appear to yield any significant effect on the attenuation of the ^1^O_2_ cell death response (Ramel et al., 2013). This was attributed to the two ^1^O_2_ generation systems differing in their signalling mechanisms.

It was found that cold/HL stress led to the accumulation of several oxidation products of β-carotene, a key carotenoid involved in the quenching of reactive oxygen species produced during photosynthesis (Ramel et al., 2012). Further *in vitro* studies showed that ^1^O_2_ was the key molecule involved in this oxidative process. Among the various oxidation products found, β-cyclocitral (BCC), a volatile organic compound, was observed to stimulate the induction of various ^1^O_2_ sensitive genes. Pre-application of BCC to WT Arabidopsis plants was able to acclimate them to a future cold/HL stress treatment (Ramel et al., 2013). BCC is thought to act as a possible signalling molecule in chloroplast-to-nuclear signalling. Putative transducers of the BCC signal include the zinc finger protein MBS1, that are important for BCC-induced acclimation to HL stress (Shumbe et al., 2017); and the TGAII/scarecrow like-14 (SCL14) transcription factors which regulate detoxification related genes for HL stress acclimation (D'Alessandro et al., 2018). Further investigations of the *Ch1* mutant have also implicated the OXI1 kinase/MAPK and salicylic acid/H_2_O_2_ signalling pathways (Shumbe et al., 2016; Beaugelin et al., 2019). These pathways appear to modulate the endoplasmic reticulum mediated stress (ER stress) and unfolded protein responses (UPR), as part of the cellular responses to high light stress (Beaugelin et al., 2020), but do not appear to involve BCC signalling.

Recently, we showed that cytosolic sources of ^1^O_2_ stimulated stress transcriptomes were correlated with cycloheximide (CHX) transcriptomes (Koh et al., 2021). CHX is a potent and irreversible inhibitor of 80S ribosomal translation (Schneider-Poetsch et al., 2010). Under CHX treatment cytoplasmic translation ceases. In the absence of continual replacement to maintain a steady state, proteins will degrade according to their respective half-lives (Li et al., 2017). Transcripts previously kept repressed by short-lived transcription factors are then induced. Using a variety of ^1^O_2_ generating systems such as Rose Bengal (RB) and DCMU, we demonstrated that ^1^O_2_ generated in the cytosol but not within the chloroplast, showed CHX-like transcriptomic elements. This could be explained by the oxidation of guanosine residues to 8-hydroxyguanosine (8-oxoG) by ^1^O_2_. The oxidized guanosine stymies 80S ribosomal translation and mimicked the action of CHX (Tanaka et al., 2007). The effect was exacerbated in proteins regulated by the proteosome that have a high turnover rate. Thus, a wave of stress transcripts that are regulated by such proteins are freed from repression and accumulate as a ^1^O_2_ -specific stress signature. For example, jasmonic acid signalling genes that are repressed by short half-life JAZ repressors were stimulated *via* ^1^O_2_ mediated translational attenuation in the *coi1* mutant, which is normally insensitive to JA (Koh et al., 2021). It is important to note that the great majority of genes do not change their expression so that a degree of specificity is achieved by limiting the response to rapidly turning over transcriptional repressors or activators without reprogramming of the entire transcriptome.

Here, we show that Pchd accumulates in both the chloroplast and cytosol of the *flu* mutant under dark incubation. Prolonged incubation in the dark led to increased Pchd accumulation in a time-dependent manner, which led to the formation of ^1^O_2_ upon exposure to light. RNA oxidation was monitored *via* the formation of oxidized guanosine (8-oxoG), and was found to accumulate proportionally with Pchd levels and light intensity. The translational competence of luciferase reporter transcripts in *flu* x IAA-LUC transgenic plants were analysed under these conditions. The results demonstrated that there was a corresponding decrease in translatability with increasing levels of 8-oxoG. Plants supplemented with ALA exhibited cytosolic accumulation of Pchd in a manner similar to the *flu* mutant, and also resulted in a strong CHX-like transcriptomic signature. In order to evaluate the contribution of ^1^O_2_ -mediated translational attenuation in various ^1^O_2_ -signalling systems such as RB, DCMU and the *flu* and *Ch1* mutants, we developed a bioinformatic method for distinguishing between cytosolic and chloroplastic sources of ^1^O_2_. We then further apply our method to analyse various stress transcriptomes and gain biological insight into the nature of ^1^O_2_ signalling in these systems.

## Materials and methods

### Plant growth conditions and treatments

*Arabidopsis thaliana* (ecotype Columbia) seedlings (2-week-old) were grown under white light in a 16-h light (120 µEm^-2^s^-1^)/8-h dark cycle at 21°C on Murashige and Skoog medium, supplemented with 1% sucrose and 0.8% (w/v) phytoagar (Invitrogen). Plants were pre-equilibrated in double-distilled water (DDW) in a Petri dish for 1 h before being transferred to 12 well plates for the various chemical treatments. For treatment of *flu* seedlings, they were placed in the dark in DDW for the time points indicated and re-exposed to light as indicated.

### Confocal microscopy and spectral imaging

Confocal microscopy analysis was carried out on 5-day old Arabidopsis seedlings. All images were taken with a Nikon A1 confocal microscope. For chlorophyll fluorescence, excitation was at 630 nm and emission was at 690 nm. All images were acquired using a 60x objective lens. Seedlings were equilibrated in DDW for 1 h in the light and subjected to the various treatments described. For WT, *flu* and *ch1* mutants, seedlings were incubated in the dark for the time points indicated, then visualized under the microscope. For ALA treatment, seedlings were incubated with 1 mM ALA (in half-strength MS + 1% sucrose, unless indicated) for the time points indicated. The laser settings were constant for all treatments and the post processing look up table was adjusted for each treatment to eliminate background fluorescence.

Confocal measurements for spectral imaging were performed using the Leica TCS SP8 with an Acousto Optical Tunable Filter (Leica microsystems CMS GmbH, Germany). A representative relevant slice was scanned using 458 nm excitation (5% power) and collection carried out in 5 nm width windows in the range of (600-700 nm). Images were acquired at a scanning speed of 8000 pixels per second with 63X oil immersion objective and image analysis was performed using Leica Application Suite software (Leica microsystems CMS GmbH).

### RNA extraction and qRT-PCR analyses

Arabidopsis seedlings (2-week-old, 7 whole seedlings per biological replicate, 3 replicates) were used for each treatment. Samples were harvested by flash freezing in liquid nitrogen and were homogenized in a shaker using glass beads. RNA was extracted from frozen tissues using a standard TRIzol extraction method (Sigma-Aldrich). DNase I (Sigma-Aldrich)-treated RNA was reverse transcribed using a high-capacity complementary DNA reverse transcription kit according to the manufacturer’s instructions (Quanta Biosciences). For qRT-PCR analysis, the SYBR Green method (KAPA Biosystems) was used on a Step One Plus platform (Applied Biosystems) with a standard fast program. qRT-PCR primers were designed in Snapgene software. All qRT-PCR primer sequences are listed in **Supplemental Dataset S1**.

### RNA oxidation and LC-MS/MS analyses

Arabidopsis seedlings (2-week-old, 7 whole seedlings per biological replicate, 3 replicates) were used for each treatment. In all downstream processing steps from plants, 4 mM of 4-hydroxy-TEMPO (Sigma-Aldrich) was used as an antioxidant to prevent spurious oxidation of RNA (Hofer & Möller, 1998). Samples were subjected to RNA digestion (2 h at 37°C with 30 units Nuclease S1 in 20 mM sodium acetate, pH 5.2, followed by 1 h at 37°C with 10 units Shrimp alkaline phosphatase in 100 mM Tris-HCl, pH 8. The reaction mixture was then filtered through a 10 kDa filtration column (Amicon) for 15 min at 14,000 rpm, 4°C, and the filtrate was collected for 8-oxoG determination. For standard curves concentrations 0, 1, 5, 10, 25 and 50 ng/mL of 8-oxoG, and 0, 1, 5, 10, 25 and 50 μg/mL of G were prepared and measured. The chromatographic separation was performed on an Acquity UPLC system (I-Class, Waters, Milford, MA, USA) with a Cortecs UPLC C18+ column (1.6 μm, 2.1×100 mm). The mobile phase was (A) 0.1% acetic acid in water, and (B) methanol. Full peak separation was achieved (RTG=4.07 min and RT8-oxo-G=5.35 min) to avoid ion-suppression of 8-oxoG by G with the following gradient program %B (min): 2(0-4), 100(7-7.5), 2(8-10). MS detection was performed on a TQ-S triple quadrupole mass spectrometer (Waters), equipped with an ESI ion source operated in the positive mode. Detections were performed in MRM mode. The MS/MS transitions selected for 8-oxoG were 300.06→168.09 (collision energy CE=17eV) and 300.06→140.05 (CE=33eV) m/z. The transitions for G were 284.1→135.2 (CE=25eV) and 284.1→152.2 (CE=65eV) m/z. The collision energies were chosen to enable simultaneous measurement of 8-oxo-G and G in the same run. MassLynx and TargetLynx software (v.4.1, Waters) were applied for the acquisition and analysis of data.

### *In vitro* translation and luciferase activity

Arabidopsis seedlings (2-week-old, 7 whole seedlings per biological replicate, 3 replicates) were used for each treatment. *In vitro* translation was performed with the Rabbit Reticulocyte Assay (Promega) according to the manufacturer’s instructions using 10 µg of RNA supplemented with 1 µl each of RNAasin Inhibitor (Promega) and Plant Protease inhibitor cocktail (Sigma) in a 50 µl final volume. The reactions were run at 30°C for 2 h in a thermocycler, and stopped by cooling the reaction to 4°C and further addition of 2 µl of 10 mM cycloheximide. Luciferase activity was measured using a luminometer (Turner Biosystems) in conjunction with Luciferase Assay Reagent (Luciferase Assay System, Promega). For luciferase measurements after *in vitro* translation the reaction was used directly without further processing.

### Protochlorophyllide fluorescence measurement

Protochlorophyllide (Pchd) accumulation was measured by imaging live plants using a fluorimeter. Arabidopsis seedlings (14-day-old) were placed in individual wells of a 24-well plate in DDW, supplemented with ALA at the concentrations and times indicated. Fluorescence was measured using a fluorimeter scanning a 3x3 array of points per well and the highest value of each well taken for analysis (Ex/Em: 440/630 nm). The means and SE of 24 whole seedlings per time point are shown.

### RNAseq and bioinformatics analyses

Libraries were prepared with the MARS-seq protocol (Jaitin et al., 2014) and sequenced using Illumina NextSeq 500 High Output v2 Kit (75 cycles). Reads were trimmed using ‘cutadapt’ (Martin, 2011) and mapped to the Araport 11 reference genome using STAR v2.4.2a (https://github.com/alexdobin/STAR/). Counting was done using HTSeq-count (Anders et al., 2015). Further analysis is done for genes having minimum 5 reads in at least one sample. Normalization of the counts and differential expression analysis was performed using DESeq2 (Love et al., 2014). Raw P values were adjusted for multiple testing using the procedure of Benjamini and Hochberg (Benjamini and Hochberg, 1995). The test samples were always compared to their respective 0 h, or untreated controls.

Microarray data was processed using the *affy*, *oligo* or open source Bioconductor *marray* R packages and normalized using *mas5* and associated packages. Transcripts were filtered for at least 2-fold up or down regulation, with a p-value cutoff of 0.05. Datasets are available in **Supplemental Dataset S2**. ROSMETER analyses were performed using the ROSMETER tool by providing fold-change and P values of the respective transcriptomes (http://app.agri.gov.il/noa/ROSMETER.php) (Rosenwasser et al., 2013). Venn diagrams were generated by: (Venny 21; http://bioinfogp.cnb.csic.es/tools/venny/). P-values were obtained by using Fisher’s Exact Test of 2x2 contingency table for each individual comparison and are represented by the numbers in parentheses.

### Translation attenuation index analysis

The reference CHX transcriptome used was derived from GSE111284. The material used to prepare the transcriptome were 14-day old Arabidopsis WT (Col-0) seedlings that had been treated with 100 µM CHX for 2 h under low light (30 µE m^-2^ s^-1^) conditions. For any gene list, we queried its respective genes against the CHX reference transcriptome. The steps of TAI analysis are the following: Up-regulated genes (>2-fold, p < 0.05) from any transcriptome are searched within the CHX transcriptome and their fold change under CHX obtained. The corresponding fold change values of the CHX genes (log_2_) are then arranged in a plot in the order of largest to smallest. The value of TAI was calculated in the following way. TAI = [Number of up-regulated genes]/[Total number of genes]. For example: A gene list of 100 genes are queried against the CHX transcriptome. If 70 genes had a log_2_ fold change value > 0, then TAI = 70/100 = 0.7.

### Accession numbers

The reference databases used here CHX, RB, *flu*, DCMU, *Ch1* are GSE111284, GSE111285, GSE111286, GSE111287, and GSE205861 respectively. The *flu*, *flu/ex1*, *flu/ex2* and *flu/ex1/ex2* transcriptomes were obtained from GEO GSE10509 (Lee et al., 2007). The *flu/ex1* and *flu/ex1/safe1* transcriptomes were obtained from GEO GSE131610 (Wang et al., 2020). The drought microarray data was obtained from ArrayExpress: E-MEXP-2377 (Mizoguchi et al., 2010). The *Ch1* (Ramel) microarray data done after dual light stress and cold treatment was obtained from Project CEA10-02_Light (Ramel et al., 2013). The other microarray data were obtained from the respective reference databases. *fc1*, *fc2* – GEO GSE71764 (Woodson et al., 2015); FR treatment – GEO GSE6169 (Page et al., 2017); *vte2* - GEO GSE4847 (Sattler et al., 2006).

## Results

### The ^1^O_2_ producing flu and Ch1 mutants have components in their transcriptomes bearing strong overlap with translational arrest induced by CHX

**Figure 1:**
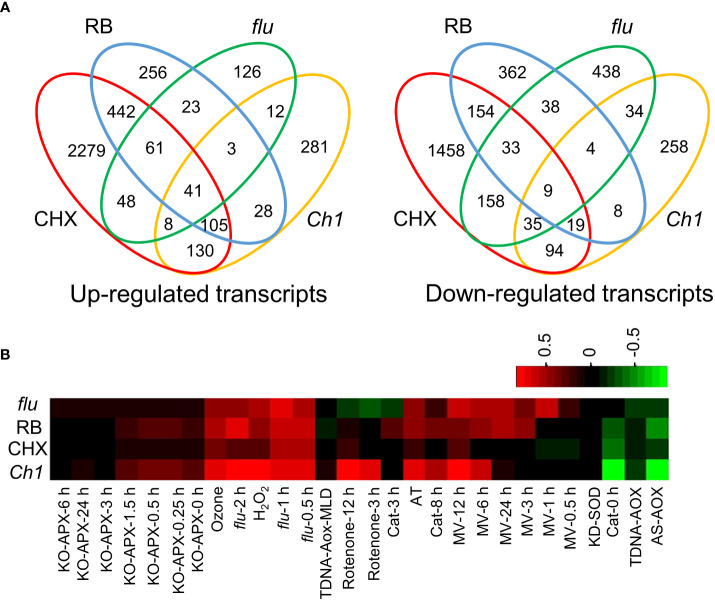
Comparison of transcriptomes of seedlings treated by RB, DCMU, cycloheximide (CHX) and from the *flu* mutant. Arabidopsis seedlings were treated with either CHX (100 µM, 2 h, 30 µE m^-2^ s^-1^) or RB (400 µM, 2 h, 30 µE m^-2^ s^-1^). The *flu* mutant was incubated in the dark for 4 h, before light exposure (30 µE m^-2^ s^-1^) for 30 min. The *Ch1* transcriptome was treated under HL/cold conditions (1200 µE m^-2^ s^-1^, 10°C, 48 h, see Ramel, 2013) Further details and the source of transcriptomes are referenced in the Methods. **(A)** Venn diagram overlaps of 2-fold induced genes of Up-regulated (left) and Down-regulated genes (right). **(B)** ROSMETER transcriptomic analysis. Transcriptomes of all significant up and down-regulated genes were analysed using the ROSMETER tool showing the correlation of gene activity with the ROSMETER databases. Red represents a positive correlation of +1, and green represents a negative correlation of -1.

The *flu* and *Ch1* mutants have been extensively studied as ^1^O_2_ generating systems although it is understood that ^1^O_2_ generation in both systems stem from different sources. The former from protochlorophyllide (Pchd) accumulation in the dark, and the latter from improperly assembled light harvesting complexes. Transcriptomes for *flu* were obtained upon transfer of plants to light from the dark (GSE111286) and for *Ch1* after dual application of light stress and cold (Ramel et al., 2013). They were compared to CHX and RB transcriptomes (Koh et al., 2021). Genes that were at least 2-fold upregulated, showed significant overlap of 49.2% (P = 2.9E-70) and 46.6% (P = 3.3E-118) with CHX and 39.9% (1.1E-106) and 29.1% (P = 1.2E-119) with RB, in *flu* and *Ch1* respectively (**Figure 1A**). Relatively, down regulated transcripts showed less but highly significantly overlap, of 31.5% (P = 2.9E-98) and 34.0% (P = 2.1E-70) with CHX and 13.5% (P = 1.7E-37) and 8.7% (P = 4.5E-14) with RB, in *flu* and *Ch1* respectively (**Figure 1A**, **Supplemental Table S1A**). The ROSMeter tool compares transcriptomes to a database of known ROS-based transcriptomes (Rosenwasser et al., 2013; Mor et al., 2014). Each has its own distinct signature; while the *flu*, and RB transcriptomes are most similar they all contain with CHX and *Ch1* mutant a common ^1^O_2_ signature. The comparisons suggest that both *flu* and *Ch1* transcriptomes contain elements of the RNA oxidation and translational arrest mechanism associated with the oxidation of RNA (**Figure 1B**). Exogenous application of H_2_O_2_ also showed similarity and such treatments have been shown to stimulate RNA oxidation as well (Hofer et al., 2005). Interestingly, some of the tested transcriptomes appeared to exhibit a negative correlation to mitochondria superoxide and H_2_O_2_ accumulation in organelles; where AS-AOX and TDNA-AOX are mitochondrial knockdown and knockout mutants of alternative oxidase, respectively (**Figure 1B**). In contrast, the chloroplast superoxide and H_2_O_2_ associated with KO-APX mechanisms appeared to be unrelated to the conditions examined.

### Cytosolic accumulation of protochlorophyllide in the *flu* mutant leads to RNA oxidation and translational arrest in a light and concentration dependent manner

**Figure 2:**
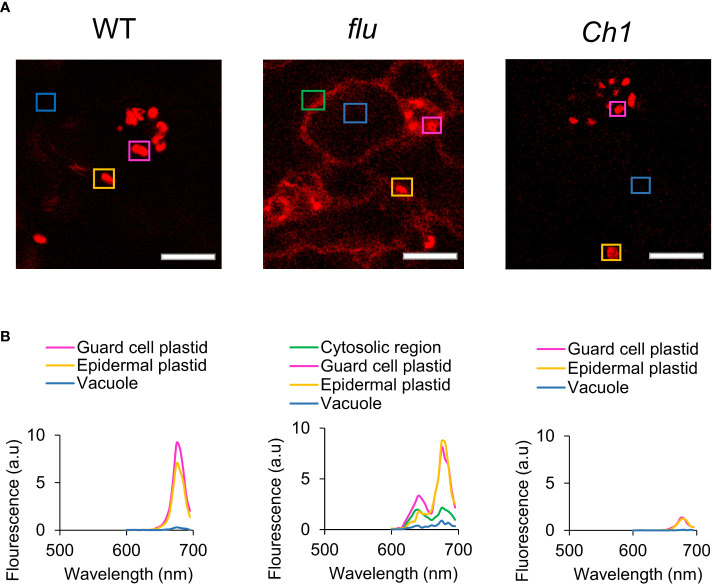
Comparison of chlorophyll fluorescence and localization in WT, *flu* and *Ch1* seedlings under dark incubation. **(A)** Spectral imaging of WT, *flu* or *Ch1* seedlings during dark incubation. WT, *flu* or *Ch1* seedlings were incubated in the dark for 4 h, then mounted in DDW for imaging as described in the Methods. Scale bar represents 5 µm. **(B)** Spectral analysis of chlorophyll fluorescence. Areas indicated by boxes in **(A)** were analyzed and the emission spectrum was plotted.

Chlorophyll biosynthesis, including the critical photoconversion of Pchd, takes place in the chloroplast, where the majority of chlorophyll biosynthetic enzymes reside. Regulated accumulation in conjunction with other components of the photosynthetic machinery is crucial as an excess of free Pchd or chlorophyll would act as disruptive photosensitizers. Pchd accumulation in the *flu* mutant was previously shown to occur only in the chloroplast (op den Camp et al., 2003). However, Pchd fluorescence of dark-incubated *flu* mutant showed cytosolic accumulation that is confirmed here [**Figure 2**; (Koh et al., 2021)]. This finding may be due to the current advances in the sensitivity of confocal technology. In comparison, dark incubated WT or *Ch1* mutant seedlings show chlorophyll localized only to the chloroplast. A precise fluorescence emission spectrum was carried out in various subcellular localizations (**Figure 2A**). It showed accumulation of two major peaks corresponding to Pchd and chlorophyll in the plastids and cytosol of the *flu* mutant, but only a single chlorophyll peak in the plastids of WT and *Ch1* (**Figure 2B**).

**Figure 3:**
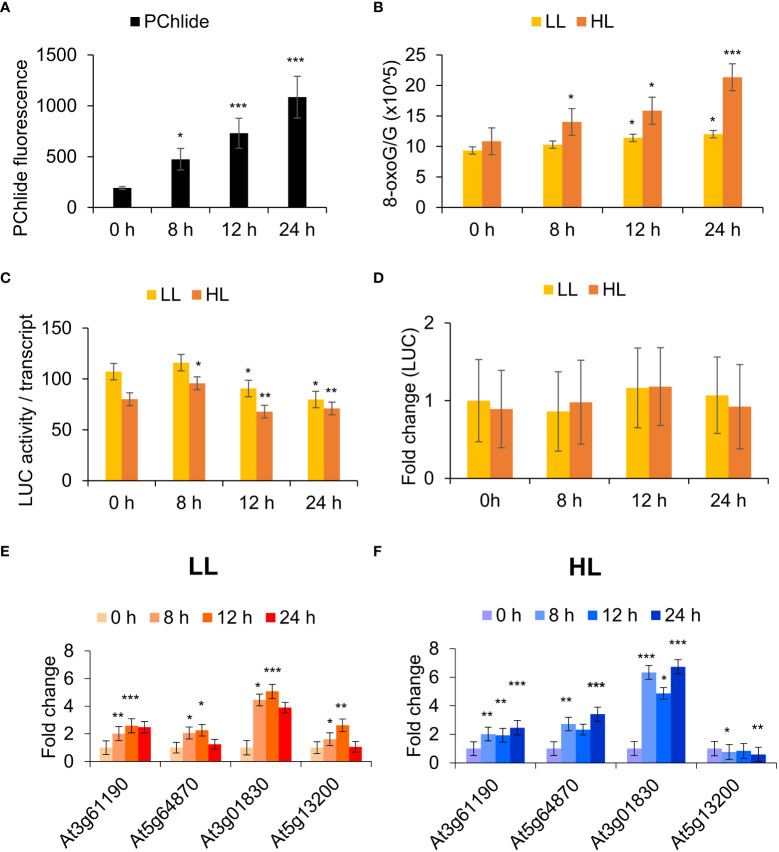
RNA oxidation in the *flu* mutant is correlated with dark incubation time and light intensity. **(A)** Protochlorophyllide (Pchd) accumulation in *flu* x UBIQ10:IAA-LUC seedlings. Fluorescence was measured in dark incubated *flu* x UBIQ10:IAA-LUC seedlings using a fluorimeter (Ex/Em: 440/630 nm). The means and SE of 24 whole seedlings per time point are shown. **(B)** RNA oxidation analysis. Arabidopsis (Col-0, *flu* x UBIQ10:IAA-LUC) seedlings were incubated in the dark for 0, 8, 12, 24 h in the dark before 30 min exposure to low light (LL, 30 µE m-2 s-1) or high light (HL, 1000 µE m-2 s-1). RNA was processed for RNA oxidation as described in the Methods. **(C)** RNA translatability analysis. RNA as in **(B)** was used for *in vitro* translation and luciferase activity analysis as described in the Methods. Values were normalized against luciferase transcript levels, and Student’s t-test performed against LL 0 h. **(D)** RNA as in **(B)** was processed as described in the Materials and Methods for qRT-PCR analysis to determine fold change in luciferase mRNA levels in the various samples using luciferase specific primers. **(E)** and **(F)** RNA as in **(B)** was extracted and processed as described in the Materials and Methods for qRT-PCR analysis. The means and SE of three replicates is shown. Student’s t-test was performed against their respective controls for significance (* - P<0.05; ** - P<0.01; *** - P<0.001). ^1^O_2_ sensitive genes: At3g61190 – Bonsai associated protein (BAP1), At5g64870 - nodulin-related protein, At3g01830 - calmodulin-related protein, At5g13200 - GRAM domain-containing protein.

The levels of Pchd can be manipulated by increasing the dark incubation time of mutant *flu* seedlings (**Figure 3A**). Guanosine residues are highly reactive to ^1^O_2_ (Wilkinson et al., 1995). To monitor this, the degree of guanosine oxidation to 8-oxoG was measured relative to guanosine (G) where the latter serves as an internal quantitative control. This ratio rises upon dark to high light transition to about 20 oxidation events for every 10^5^ residues (**Figure 3B**). Assuming an average transcript length of about 1500 bp for Arabidopsis, this seemingly low rate would still lead to approximately one oxidation event in 7.5% of the transcripts. That level can have profound effects on rapidly turning over proteins that need constant replenishment by transcript translation to maintain their steady state.

IAA-LUC fusions produce constitutive levels of IAA-luciferase mRNA and can be used to precisely measure changes in mRNA functionality by comparing the translational competence of its RNA. To precisely measure the effect of 8-oxoG accumulation in the *flu* mutant, a *flu* x UBIQ10:IAA-LUC transgenic line was constructed. The effect of dark incubation time and subsequent exposure to light on the translational competence of constitutively expressed luciferase transcripts was then measured in a reticulocyte translation system. We observed that the relative translational competence of luciferase transcripts showed a significant decrease at 12 and 24 h dark of incubation time and was sensitive to higher light intensity (**Figure 3C**). The levels of LUC mRNA as measured by qPCR were used to normalise the luciferase activity levels. They remained relatively unchanged in all treatments (**Figure 3D**). Using a ^1^O_2_ -specific gene set previously validated to be responsive to RNA oxidation and translational arrest, we observed that increased dark incubation time and light intensity led to a general increase in expression of these genes (**Figures 3E, F**). **Figure 3E** shows that at later times the gene activity of the ^1^O_2_ marker genes is decreasing (i.e. at 24 h, rather than continuing to increase. This could be due to cross regulation by compensatory mechanisms e.g. increased RNA synthesis in LL (low light) conditions; whereas these mechanisms are not sufficient to compensate under (HL) high light intensities (**Figure 3F**). Thus, we show that Pchd accumulates proportionally to the period of dark incubation, and this Pchd then generates ^1^O_2_ and RNA oxidation in a manner dependent on light intensity. Following from this, the induction of ^1^O_2_-signature gene expression in the *flu* mutant is correlated with the oxidation of RNA and the level of its translational competence.

### ALA treatment in the dark leads to cytosolic accumulation of chlorophyll precursors in the cytosol, but is not modulated by the executor pathway

**Figure 4:**
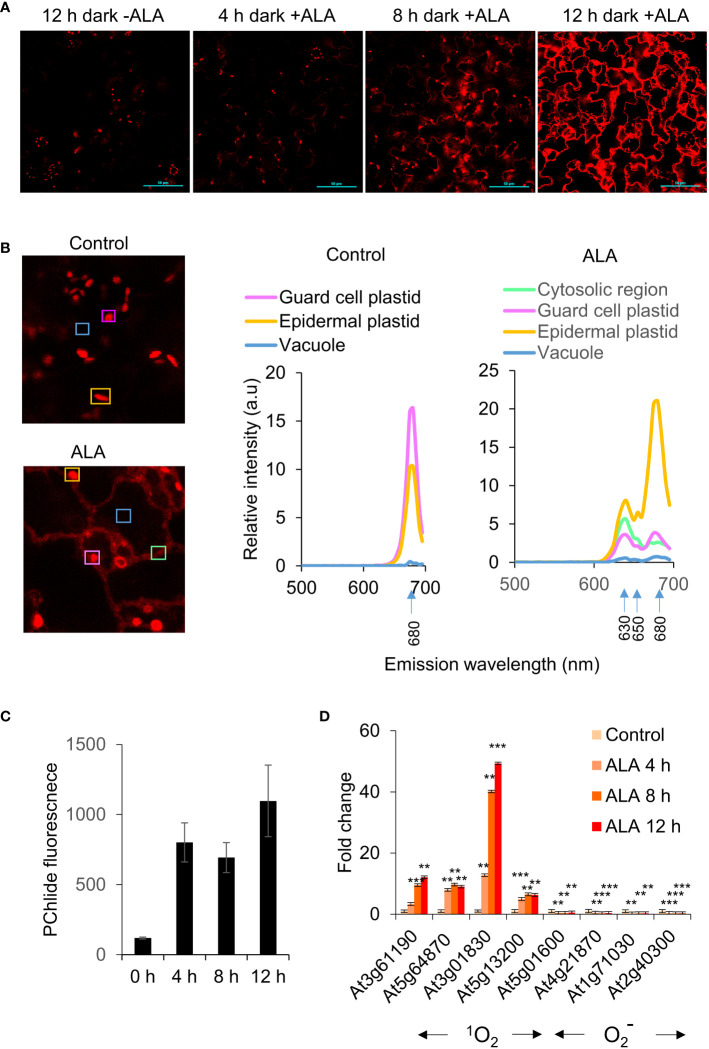
ALA feeding leads to extrachloroplastic accumulation of chlorophyll. **(A)** Accumulation of chlorophyll in extrachloroplastic regions under ALA treatment. Arabidopsis (WT, Col-0) seedlings were incubated with 1 mM ALA (in MS) in the dark for 12 h, and then mounted for visualisation as described in the Methods. Scale bar represents 50 µm. **(B)** (Left) Spectral imaging of Arabidopsis (WT, Col-0) seedlings were treated with DDW or 1 mM ALA (in DDW) and incubated in the dark for 2 h, and then mounted for visualisation by confocal microscopy as described in the Methods. Scale bar represents 50 µm. (Right) The areas indicated by boxes were scanned for spectral analyses as shown on the right. **(C)** Protochlorophyllide (Pchd) accumulation in ALA treated seedlings. Fluorescence was measured in WT seedlings (WT, Col-0) treated with 1 mM ALA (in MS) and incubated in the dark for 0, 4, 8, 12 h. Pchd fluorescence was measured using a fluorimeter (Ex/Em: 440/630 nm). The means and SE of 24 whole seedlings per time point are shown. **(D)** Arabidopsis (WT, Col-0) seedlings were incubated with 1 mM ALA (in MS) in the dark for 0, 4, 8, 12 h in the dark before 30 min exposure to low light (30 µE m^-2^ s^-1^). RNA was extracted and processed as described in the Materials and Methods for qRT-PCR analysis. The means and SE of three replicates is shown. Student’s t-test was performed against their respective controls for significance (** - P<0.01; *** - P<0.001). ^1^O_2_ sensitive genes as in Figure 3E. ${\text{O}}_{2}^{-}$ sensitive genes: At5g01600 – Ferritin 1 (FER1), At4g21870 - heat shock protein family, At1g71030 - myb family transcription factor, At2g40300 - ferritin-related (FER4).

δ-aminolaevulinic acid (ALA) is an early precursor of chlorophyll (Granick, 1959). As the *flu* mutant is defective in the negative feedback regulation of ALA synthesis in the dark, we examined to what extent supplementation of the media with ALA could phenocopy the *flu* mutant. WT Arabidopsis seedlings incubated with ALA in the dark were observed to accumulate cytosolic fluorescence in a time dependent manner (**Figures 4A, C**). Analysis of the fluorescence emission spectrum of ALA treated plants showed twin peaks of Pchd and chlorophyll in the plastids as well as the cytosol. The untreated plants subject to the same period of dark incubation had only a single chlorophyll peak and no fluorescence in the cytosol could be detected (**Figure 4B**). qPCR analysis of ALA treated plants exposed to light showed a strong time-dependent induction of transcripts from ^1^O_2_ sensitive genes, but not from superoxide ( ${\text{O}}_{2}^{-}$ ) sensitive genes (**Figure 4D**). The results indicate that dark incubation with ALA mimics the *flu* mutant in showing accumulation of chlorophyll precursors in the cytosol and the induction of a ^1^O_2_ transcriptome upon exposure to light.

The executor (Ex) pathway was previously established as a genetic model that abrogated the cell death phenotype of the *flu* mutant. Under 16 h light/8 h dark growth conditions, the *flu* mutant develops necrotic lesions and severely impaired growth. In contrast, the *flu/ex1* mutant displayed normal growth similar to WT and a distinct lack of lesions (Wagner et al., 2004). Another mutation, *ex2*, was observed to be unable to abrogate cell death in the *flu/ex2* mutant, but was able to enhance the protective effect of *ex1* in the *flu/ex1/ex2* triple mutant (Lee et al., 2007). However, Pchd and ^1^O_2_ accumulation was reported to be similar in the *flu*, *flu/ex1*, *flu/ex2* and *flu/ex1/ex2* mutants (Kim et al., 2012).

**Figure 5:**
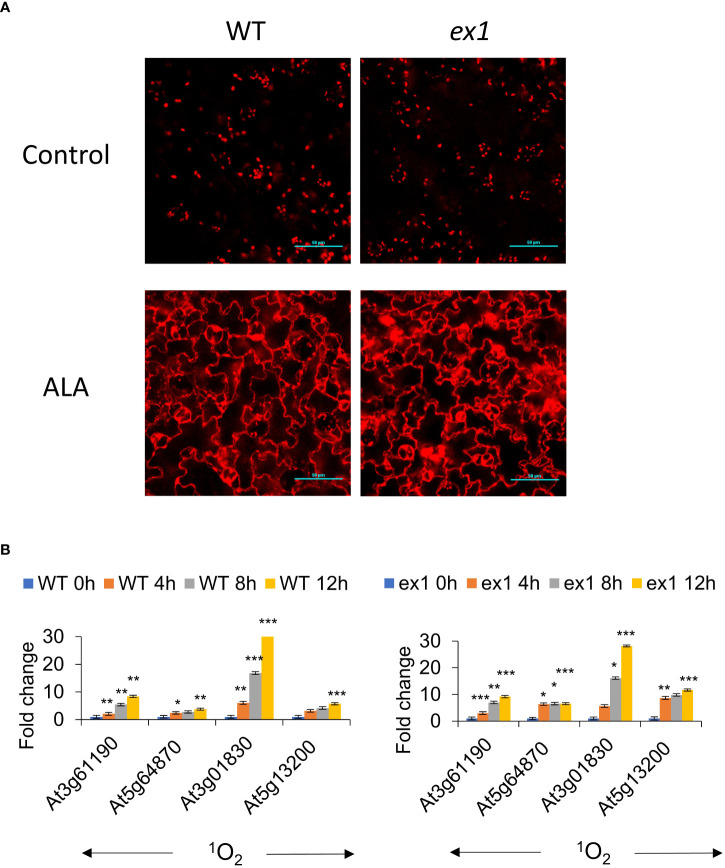
Extrachloroplastic accumulation of chlorophyll is not regulated by the executor pathway under ALA treatment. **(A)** Accumulation of chlorophyll in extrachloroplastic regions under ALA treatment. Arabidopsis WT and *ex1* seedlings were incubated with 1 mM ALA (in MS) in the dark for 12 h, and then mounted for visualisation by confocal microscopy. Ex/Em: 630/690 nm. Scale bar represents 50 µm. **(B)** Arabidopsis WT and *ex1* seedlings were treated with 1 mM ALA (in MS) and incubated in the dark for 0, 4, 8, 12 h in the dark before 30 min exposure to low light (30 µE m^-2^ s^-1^). RNA was extracted and processed as described in the Materials and Methods for qRT-PCR analysis. The means and SE of three replicates is shown. Student’s t-test was performed against their respective controls for significance (* - P<0.05; ** - P<0.01; *** - P<0.001).

**Figure 6:**
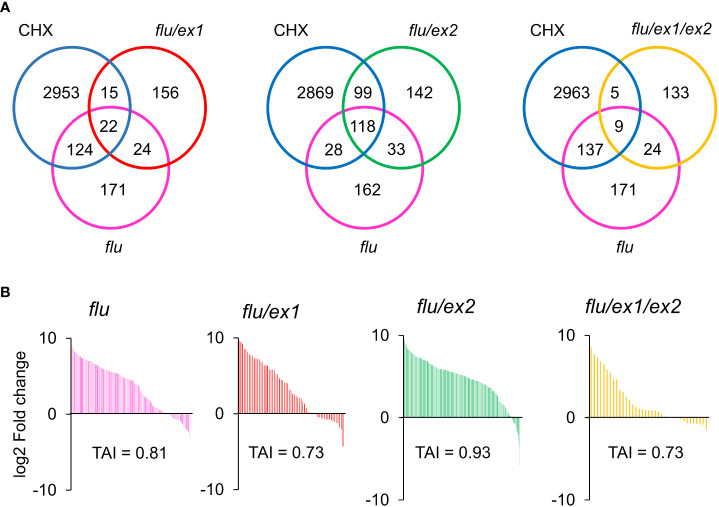
Venn diagram and Translational Attenuation Index analysis of *flu, flu/ex1, flu/ex2, flu/ex1/ex2* transcriptomes. **(A)** Venn diagram overlaps of 2-fold up-regulated (p < 0.05) genes of CHX, *flu*, *flu/ex1*, *flu/ex2*, *flu/ex1/ex2*. **(B)** Translational Attenuation Index analysis of CHX, *flu*, *flu/ex1*, *flu/ex2* and *flu/ex1/ex2* induced transcripts. Genes from *flu*, *flu/ex1*, *flu/ex2*, *flu/ex1/ex2* transcriptomes as in **(A)** were screened against a dataset of cycloheximide treatment and their expression values under cycloheximide treatment are displayed accordingly. The data from CHX, *flu*, *flu/ex1*, *flu/ex2* and *flu/ex1/ex2* were obtained from public databases. (CHX – GSE111284; *flu*, *flu/ex1*, *flu/ex2* and *flu/ex1/ex2* – GSE10509).

In order to investigate the role of the executor pathway in the cytosolic accumulation of Pchd, ALA treatment was used to generate chlorophyll precursors in the WT, *ex1, ex2* and *ex1/ex2* mutants. We noted that the rate of Pchd accumulation in bulk tissue was similar between WT, *ex1*, *ex2* and *ex1/ex2* mutants under ALA treatment (**Supplemental Figure S1**). Next, we observed that in both the WT or in the *ex1* mutant significant Pchd fluorescence accumulated in the cytosol under ALA treatment (**Figure 5A**). Furthermore, ^1^O_2_ signature genes were all similarly induced in the WT and *ex1* mutants after transition to light (**Figure 5B**). It is also important to note that in the longer dark incubation times the induction of these genes was much greater than in the *flu* mutant (compare **Figures 3E**, **4D**, **5B**). This is likely due to greater accumulation of Pchd in the cytoplasm (compare images in **Figure 2A**, *flu* and **Figure 4A**, ALA). We examined published transcriptomes of *flu*, *flu/ex1*, *flu/ex2* and *flu/ex1/ex2* mutants subjected to dark-light shifts by comparing them to the CHX transcriptome. Both *flu* and *flu/ex2* mutants showed significant overlap with CHX, that was reduced in the *flu/ex1* and especially in the *flu/ex1/ex2* combinations (**Figure 6A**). In contrast, feeding by ALA leads to more substantial accumulation that cannot be inhibited by the *executor* mutations as shown in **Figures 5A, B**.

A recent study described a grana localised protein, SAFEGUARD1 (SAFE1), that appeared to play an important role in protecting the chloroplast against ^1^O_2_-mediated toxicity in the *flu* mutant, but independently of the EX1 pathway (Wang et al., 2020). The absence of this protein in the *flu/ex1/safe1* triple mutant led to significant symptoms of ^1^O_2_-mediated toxicity that is produced in the *flu* mutant, but is typically attenuated in the *flu/ex1* mutant. We compared published transcriptomes of the *flu/ex1* and *flu/ex1/safe1* mutants subject to dark-light shifts against the CHX and RB transcriptomes. We observed that the *flu/ex1* transcriptomes showed approximately 32.5% (p = 3.9 E-11) and 24.2% (p = 1 E-17) overlap with CHX and RB respectively, which was further increased to 77.7% (p = 0) and 46% (p = 0) respectively in the *flu/ex1/safe1* mutant (**Supplemental Figure S2**). Furthermore, it was also reported that the dark-light shift led to chloroplast leakage in this mutant, whereas plastid integrity was maintained in the *flu/ex1* mutant under the same conditions (Wang et al., 2020). These results support the notion that SAFE1 is an important component for the maintenance of plastid integrity preventing leakage of photodynamic components to the cytoplasm.

### Translational attenuation index analysis of transcriptomes can be used to differentiate between cytosolic and chloroplastic sources of ^1^O_2_

Comparison of experimental transcriptomes to the CHX transcriptome can provide an estimation of the degree of translational repression due to ^1^O_2_ production in the cytoplasm. To carry this out, genes from various transcriptomic experiments were subjected to a 2-fold expression threshold and p<0.05 significance cut-off. The selected genes were compared to CHX fold changes that is used as a common reference for all comparisons. The percentage of genes that are upregulated and the relative amplitude of their fold-change provides insight into the ^1^O_2_-responsiveness of the experimental transcriptome. We can use these properties to assign a simple quantitative index which we will term here as the Translational Attenuation Index (TAI), where the highest theoretical value that occurs in complete overlap would be 1 (See Methods).

The TAI values were computed for the *flu* (0.81); *flu/ex1*(0.73); *flu/ex2* (0.93) and *flu/ex1/ex2* (0.73) transcriptomes (**Figure 6B**). While the *flu/ex1* and *flu/ex1/ex2* mutants showed a decreased TAI (**Figure 6B**), the *flu/ex2* mutant showed a higher TAI than even the *flu* mutant. These values are consistent with previous analyses showing that *ex1* can mitigate the *flu* stress phenotype, while *ex2* alone appears to enhance it (Lee et al., 2007). In any case, the executor mutations appear to only attenuate the effect of *flu*, suggesting that the presence of cytosolic ^1^O_2_ or its effect may be reduced but not completely abrogated by the executor pathway, in agreement with the results of the application of ALA. In addition, TAI analysis for the *flu/ex1* and *flu/ex1/safe1* transcriptomes obtained by a separate group showed a TAI value of 0.57 for *flu/ex1*, while the TAI value of the *flu/ex1/safe1* transcriptome was further increased to 0.90 (**Supplemental Figure S2**), consistent with the observation that plastid integrity was compromised in this mutant under a dark-light shift (Wang et al., 2020), which may lead to leakage of chlorophyll metabolites into the cytoplasm.

### Combined cold/HL stress in the Ch1 mutant leads to significant induction of ^1^O_2_ activity and translational attenuation

Previous observations showed that the *Ch1* mutant under combined cold/HL stress conditions produced significantly greater amounts of ^1^O_2_ and lipid peroxidation compared to WT plants (Ramel et al., 2013). The *Ch1* mutant under combined cold/HL stress showed a strong ^1^O_2_ response, with significant overlaps of *Ch1* with CHX, RB and the *flu* mutant (**Figure 1**). It was established that the *Ch1* mediated photooxidative stress response did not appear to be dependent on the executor pathway, as *Ch1/ex1* double mutant plants were indistinguishable from *Ch1* plants under cold/HL stress (Ramel et al., 2013). Venn diagram analysis using publicly available *Ch1* transcriptomes show a strong overlap of the *Ch1* mutant to CHX (46.6%, p = 3.3E-118) and RB (29.1%, p = 1.2E-119), suggesting the possibility of a significant common effect on cytosolic translation (**Supplemental Figure S3**, **Supplemental Table S1A**). Consistent with the overlap, the *Ch1* mutants under cold/HL showed a relatively high TAI score (TAI = 0.69, **Supplemental Figure S3**); indicating that the combined cold/HL stress likely impacted on translational activity.

Interestingly, observations showed that cold/HL treatment caused chloroplast rupture and leakage of chloroplast contents into the cytosol (Kim et al., 2012). Therefore, under those conditions, the source of the ^1^O_2_ effect may stem from released photodynamic chlorophyll pigments. For example, green sections of the *var2* mutant manifested chloroplast rupture and cell death whereas albino sections do not (Kim et al., 2012). In a similar manner, the ferrochelatase 2 (FC2) protein is responsible for the insertion of iron into protoporphyrin IX to form protoheme. In its absence (i.e. *fc2)*, chlorophyll biosynthesis is disrupted and chloroplasts showed progressive degradation as a function of ^1^O_2_ stress (Fisher et al., 2021).

**Figure 7:**
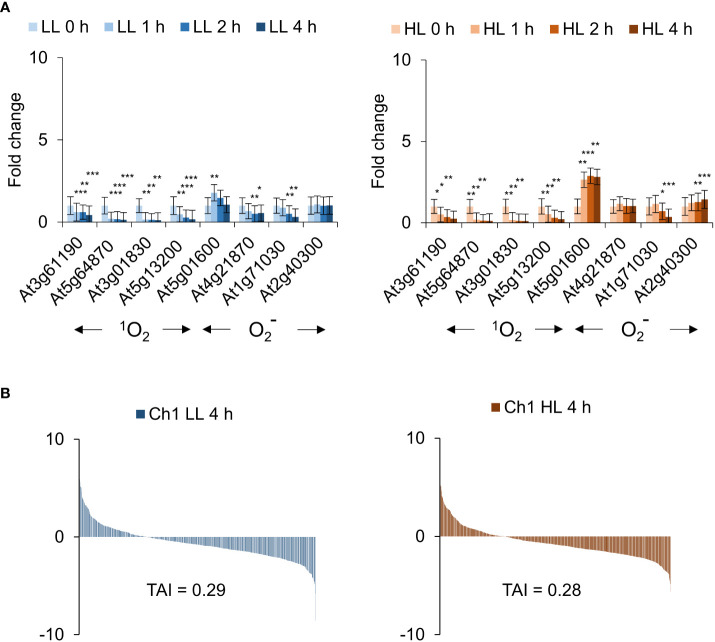
*Ch1* seedlings do not present cytosolic ^1^O_2_ signatures under low and high light treatment. **(A)** Arabidopsis *Ch1* seedlings were exposed to low light (30 µE m^-2^ s^-1^) or high light (1000 µE m^-2^ s^-1^) for 0, 1, 2, 4 h). RNA was extracted and processed as described in the Materials and Methods for qRT-PCR analysis. The means and SE of three replicates is shown. Student’s t-test was performed against their respective controls for significance (* - P<0.05; ** - P<0.01; *** - P<0.001). **(B)** Translational Attenuation Index analysis of *Ch1* induced transcripts under low light (30 µE m^-2^ s^-1^) or high light (1000 µE m^-2^ s^-1^) conditions after 4 h.

The mutant *Ch1* was also examined in moderate conditions without cold for 0, 1, 2, 4 h in LL and HL. Interestingly in this case, ^1^O_2_ responsive genes were not induced; indeed, they appear to be repressed (**Figure 7A**). In contrast, superoxide ( ${\text{O}}_{2}^{-}$ ) responsive genes were induced in a light and time dependent manner (**Figure 7A**). To extend these observations, RNAseq analysis was carried out. In the absence of cold, only a small overlap of less than 10% between *Ch1* LL/HL transcriptomes and that of *Ch1* under cold/HL stress was obtained. Furthermore, in the absence of cold, moderate overlaps of 20-30% with DCMU and *Ch1* were observed (**Supplemental Tables S2**, **S3**) and low TAI values were obtained (LL TAI = 0.29; HL TAI = 0.28; **Figure 7B**).

As β-cyclocitral (BCC) is also thought to be a signalling molecule involved in transducing ^1^O_2_ signalling in the *Ch1* mutant, we performed Venn diagram analysis using publicly available *Ch1* (cold/HL) and BCC transcriptomes showed that the *Ch1* mutant when carried out with cold treatment had a strong overlap with CHX (46.6%, p = 3.3E-118) and RB ((29.1%, p = 1.2E-119), indicating a significant effect on cytosolic translation (**Supplemental Table S1A**). Application of BCC showed a similar but slightly weaker overlap with CHX (31.4%, p = 4.4E-23) and RB (24.2%, p = 1.2E-41), while demonstrating a strong overlap with *Ch1* (48.8%, p = 1.1E-164) (**Supplemental Table S1A**). In contrast, when compared to *Ch1* LL and HL transcriptomes carried out here without additional cold treatment, the BCC transcriptome showed significantly weaker overlaps of between 4-7% for LL and 10-15% for HL (**Supplemental Table S4**). Thus, the increased light intensity with cold treatment stimulates a certain level of BCC production. These results suggest that in the case of *Ch1*, application of multiple stresses such as cold/HL treatment may result in leakage from chloroplasts causing relatively high TAI correlations and elevated BCC levels (compare **Supplemental Figure S3** and **Figure 7B**). In contrast, under conditions where chloroplast integrity is maintained and ^1^O_2_ production limited to the chloroplast, ^1^O_2_ can elicit some form of BCC-mediated retrograde signalling, but does not affect cytosolic translation and thus does not stimulate ^1^O_2_ sensitive genes.

### Transcriptome analysis can be used to gauge the contribution of cytosolic versus chloroplastic ^1^O_2_ under various stress conditions

**Figure 8:**
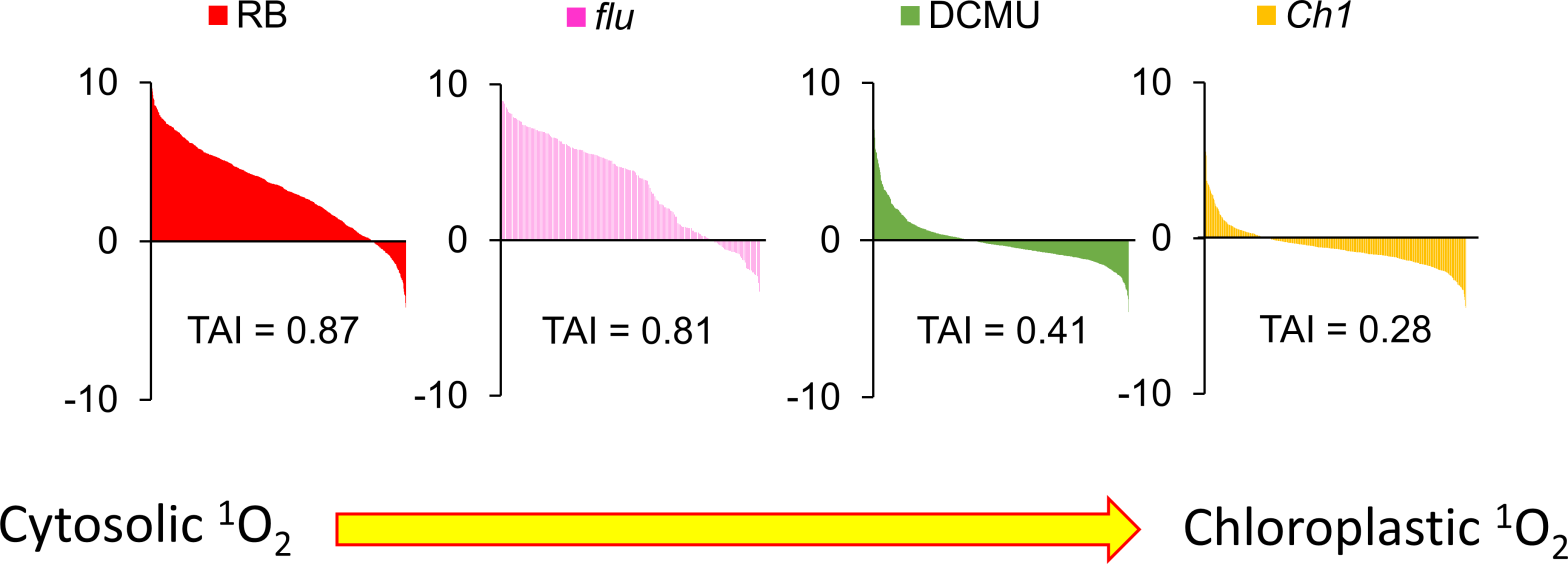
Cytosolic but not chloroplastic ^1^O_2_ production are correlated with CHX-induced transcriptomic responses. Translational Attenuation Index analysis of RB, *flu*, DCMU and *Ch1* transcripts. Genes were obtained from various transcriptomic experiments in the literature for the specific treatments and subjected to a 2-fold expression threshold and p<0.05 significance cutoff. Each list of >100 genes were then screened against a dataset of cycloheximide treatment and their expression values under cycloheximide treatment are displayed accordingly. The data here were obtained from public databases (CHX – GSE111284; RB -GSE111285; *flu* - GSE10509; DCMU - GSE111287; *Ch1* - GSE205861).

The transcriptome of CHX was compared to other ^1^O_2_ generating systems known to be localized to the cytoplasm or to the chloroplast. For example, the herbicide diuron (DCMU) blocks the electron transfer between QA and QB of PSII causing photoinhibition *via* production of ^1^O_2_ in the chloroplast (Fufezan et al., 2002). RB was shown previously to localize mainly to the plasma membrane, and elicit detectable cytosolic ^1^O_2_ production (Koh et al., 2016). As shown in **Figure 8**, the ordering of the various transcriptomes based on their TAI values: RB, *flu*, DCMU, *Ch1*, are consistent with their putative cellular locations; placing RB and *flu* in one class (cytosol) and DCMU and *Ch1* in another (chloroplast).

It was of interest to analyse additional mutations known to cause ^1^O_2_ production and examine their localization based on similarity to the CHX transcriptome. The *fc2* mutant, in a manner similar to *flu*, accumulates Pchd in the dark and displays a *flu*-like phenotype (Woodson et al., 2015). Interestingly, its light stress transcriptome shows significant overlaps with CHX, RB and *flu* transcriptomes (25-30%; TAI = 0.66; **Supplemental Table S1A** and **Supplemental Figure S4**). The weaker mutant *fc1*, that is also a ferrochelatase mutant, accumulates predominantly Protoporphyrin IX in the dark that is a less potent photosensitiser (Scharfenberg et al., 2015). The mutant *fc1* shows a weaker yet significant overlap with CHX, RB and *flu* compared to *fc2*, (TAI = 0.51; **Supplemental Table S1A** and **Supplemental Figure S4**). As noted above, progressive chloroplast degradation occurred in the light as a function of ^1^O_2_ stress in *fc2* (Fisher et al., 2021). Thus, the origin of some components of nuclear signalling in ferrochelatase mutants may emanate from photosensitizers stimulating direct cytosolic oxidation in a manner similar to the *flu* mutant.

Growth in Far-Red (FR) light has been shown to cause the accumulation of Pchd through its ability to stimulate protochlorophyllide biosynthesis, but not allowing for its efficient photoconversion to chlorophyll (Page et al., 2017). Analysis shows that transcriptomes of WT plants exposed to FR treatment and then exposed to white light overlap with CHX (28.1%, p-value = 1.3E-25) and RB (25.3%, p-value = 8.4E-68), respectively (**Supplemental Table S1A**). TAI analysis yields values of TAI = 0.62, suggestive of the generation of cytosolic ^1^O_2_ features in this case as well (**Supplemental Figure S4**).

## Discussion

### Transcriptomic signatures are correlated with ^1^O_2_ localization

^1^O_2_ is a potent ROS. Due to the ease of quantitative measurement, the oxidation of unsaturated fatty acids by ^1^O_2_ is well established. However, compared to unsaturated fatty acids, the oxidation of guanosine (G) residues is about 100-fold more reactive towards ^1^O_2_ (Wilkinson et al., 1995). When produced in the cytoplasm, ^1^O_2_ causes the oxidation of RNA and attenuation of cytoplasmic ribosomal translation. This results in decreased levels of short-lived repressor proteins, and stimulates the de-repression of the genes that they control (Koh et al., 2021). From our analysis here and previously, we observed that many ^1^O_2_ signalling transcripts were highly correlated with CHX treatment, supporting the hypothesis of ^1^O_2_ promoting translational attenuation (Koh et al., 2021). Here, we compare various light-dependent stress transcriptomes to that of the CHX transcriptome, so as to discern if cytoplasmic production of ^1^O_2_ is involved.

**Figure 9:**
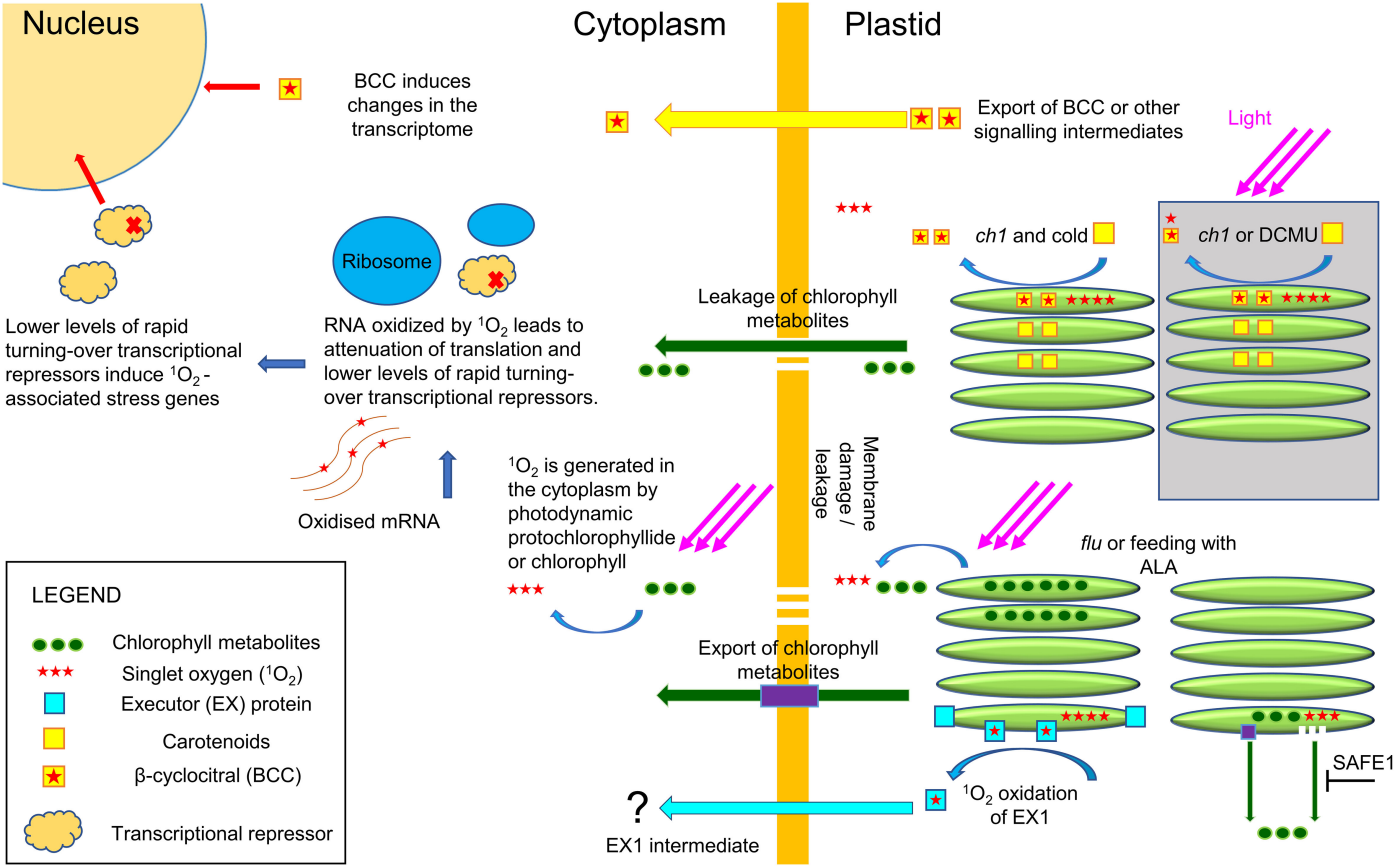
Plastid and cytoplasmic origins of ^1^O_2_. The *flu* mutant in the dark, or application of ALA, leads to the accumulation of chlorophyll metabolites and production of ^1^O_2_ in the cytoplasm in the light. It is currently unknown if the export of these metabolites occurs *via* specialized transport proteins or *via* membrane leakage. In the cytoplasm, ^1^O_2_ efficiently and selectively oxidizes guanosine residues in RNA that then inhibit translation lowering the levels of rapidly turning over repressors of transcription. The decrease in repressors will initiate the ^1^O_2_ transcriptome signature that resembles the transcriptome of CHX, an inhibitor of 80S ribosome translation. The EX1 mutant pathway has been shown to attenuate *flu*-mediated cell death, but how EX1 signals are transported outside the chloroplast and elicits nuclear signaling is currently unknown. In the light, the *ch1* mutant or application of DCMU will induce ^1^O_2_ locally in the chloroplast and initiate a transcriptome response that is distinct from cytoplasmic ^1^O_2_ (area in grey). It has been shown that BCC can serve as a signaling intermediate in the *Ch1* mutant and HL stress, but its mechanism of action is currently unknown. However, under conditions of high light and cold the increased levels of ^1^O_2_ initiate chloroplast leakage and production of ^1^O_2_ in the cytoplasm leading to a ^1^O_2_ transcriptome signature of cytoplasmic origin.

Utilizing well known models that generate ^1^O_2_ signalling such as RB, DCMU and the *flu* and *Ch1* mutants, TAI analyses provide a unified basis for examining their properties. For instance, RB preferentially localizes to the plasma membrane, while DCMU is a competitive analogue of plastoquinone targeted to the chloroplast. In each case ^1^O_2_, due to its short half-life, is generated locally and will biologically interact in its specific locality. RB and *flu* scored high in the TAI scale indicative of cytoplasmic ribosomal attenuation, while DCMU and *Ch1* (without dual stress of cold treatment) had the lowest scores (**Figure 8**). While the bioinformatic results of RB, DCMU and *Ch1* were anticipated, the cytoplasmic accumulation of *flu* and its concomitant high TAI score as shown here were not. Indeed, as Pchd was thought to localize only to the chloroplast its influence *via* photodynamic production of ^1^O_2_ on cellular expression was thought to occur exclusively through a retrograde signalling system from the chloroplast to the nucleus (op den Camp et al., 2003; Wagner et al., 2004). However, the observation that Pchd accumulates in the cytoplasm as well, illustrates how ^1^O_2_ is produced in the cytoplasm and can oxidize mRNA. Thus, while parallel retrograde pathways may exist, the oxidation of mRNA provides a mechanistic approach. A graphical summary of the key concepts discussed in the paper is provided in **Figure 9**.

Another system that stimulates Pchd accumulation is FR treatment (Sperling et al., 1997; McCormac and Terry, 2004). Continual exposure to FR light leads to an increase in Pchd. This is consistent with analysis of the FR light treatment transcriptome that exhibits a significant overlap with CHX (**Supplemental Table S1A** and **Figure S4**). Likewise, the *fc2* mutant conditionally accumulates photodynamic protoporphyrin IX and Pchd in the dark (Woodson et al., 2015). The mutant was found to rapidly bleach and die under a 16 h light/8 h dark light cycle, but not under continuous light, similar to the *flu* mutant. The *fc2* mutant upon dark-light transition showed similarity with the CHX transcriptome (**Supplemental Table S1A** and **Supplemental Figure S4**). Based on this analysis, one may expect that Pchd accumulates in the cytoplasm in these cases as well.

Mutants of EX1 and EX2 attenuate effects of the *flu* mutant. They localize to the grana margins of the thylakoid stacks in the chloroplast (Wang et al., 2016), and were found to interact with several members of the chloroplast quality control pathway, as well as the import or export machinery (Fang et al., 2022). The ^1^O_2_-mediated oxidation under dark-light transition, and subsequent proteolysis of the EX1 protein by FtSH2 was proposed to produce a retrograde signal in the nucleus (Dogra et al., 2019). The executor pathway was shown to attenuate, but not negate, the effects of the dark-light shift in the *flu* mutant (Wagner et al., 2004). Indeed as was shown, the inhibitory effect of executor was overwhelmed by longer periods of dark incubation or high light intensity (Wang and Apel, 2018). A scenario whereby the EX1 pathway impacts on the cytoplasmic proteasome may be one way in which EX imparts retrograde signalling. Indeed, inhibition of the proteasome was found to attenuate the ^1^O_2_-induced transcriptome response (Koh et al., 2021).

Feeding of the chlorophyll precursor, ALA, in plants with the *ex1* mutations had no effect on the accumulation of Pchd; either in bulk tissue measurements or by visualization of Pchd in the cytoplasmic region (**Figure 5A**, **Supplemental Figure S1**). Also, the stimulation of ^1^O_2_ signature genes showed no significant difference between the ALA treated WT or *ex1* (**Figure 5B**). Thus, the executor mutations are likely to regulate ^1^O_2_ signalling only under limiting conditions of Pchd accumulation as demonstrated in **Figure 6**. With higher levels of Pchd, such as occurs in application of ALA or longer dark incubation of the *flu* mutant *ex1* is less effective (**Figure 3** and **Figure 5**). The grana localized protein SAFE1 was found to be important in preventing the degradation of the grana margins under conditions where ^1^O_2_ is produced, and thus acts as an important buffer under physiological levels of ^1^O_2_ toxicity (Wang et al., 2020) Degradation of the thylakoids could lead to the release of photodynamic molecules, that would generate ^1^O_2_ under light.

### Environmental stresses, scavenger availability, and chlorophyll leakage as parameters in ^1^O_2_ mediated signalling

When bound by the photosynthetic machinery chlorophyll is protected by proximal antioxidants such as carotenoids. However, in the event in which photodynamic chlorophyll intermediates accumulate (as in the *flu* mutant or ALA treatment) or where photosynthesis is disturbed (as in the *Ch1* mutant or DCMU application), potent and dangerous ^1^O_2_ is produced. This is especially exacerbated in the presence of additional stresses e.g. cold or high light or their combinations (Mittler, 2006; Bratt et al., 2016). The discovery of extraplastidic RNA targets for ^1^O_2_ and the possibility of chlorophyll leakage or export from the chloroplast adds direct mechanistic understanding to its effect on gene induction (Koh et al., 2021).

Bioinformatic comparisons of the *Ch1* mutant are a case of interest as it shows a transition from a transcriptomic character solely defined by a chloroplastic localization to one which is commiserate with cytoplasmic localization. When conditions were moderate, ^1^O_2_ production was limited to the chloroplast and its integrity was maintained. Under these conditions, TAI scores were low and comparable to DCMU as both are localized to the chloroplast (**Figure 7** and **8**). Indeed, ^1^O_2_ signature transcripts in the *Ch1* mutant appeared to be repressed, while the superoxide/hydrogen peroxide signature transcripts, indicative of perturbation in photosynthetic flux, increased with the light intensity and its duration (**Figure 7A**). In contrast, when the *Ch1* mutant was challenged with simultaneous cold and HL conditions they showed significant lipid peroxidation (Ramel et al., 2013); and as shown here the transcriptome yielded a significant TAI score. The latter conditions may stimulate chloroplast rupture and leakage of chloroplastic photodynamic metabolites into the cytoplasm and stimulate ^1^O_2_ production in a manner similar to *flu* and RB. Alternatively, the activity of lipoxygenases on free fatty acids has been shown to be an additional source of ^1^O_2_ (Chen et al., 2021); these elements may be freed from the chloroplast during stress.

Chloroplast leakage in dehydration stress conditions in the light was also shown to occur concomitantly to a rise in RNA oxidation and ^1^O_2_ signature gene expression (Koh et al., 2021). Examination of published transcriptomes from dehydration treated leaves yielded a 58.3% overlap with CHX and a TAI value of 0.90 (**Supplemental Figure S3C, D**). Thus, once photodynamic material is released to the cytoplasm, ^1^O_2_ can disrupt ribosomal translation stimulating signature genes for translational arrest (Koh et al., 2021). Furthermore, chlorophyll degradation products can accumulate under conditions of starvation, senescence and pathogenesis (Pružinská et al., 2007; Hörtensteiner and Kräutler, 2011) that can also serve as a source of ^1^O_2_. Interestingly, the infestation of Arabidopsis by the beet cyst nematode *Heterodera schachtii* was found to lead to decreased chlorophyll levels, and an increase in 8-oxoG content (Labudda et al., 2018). In this manner, ^1^O_2_ generating systems that are normally sequestered in the plastid are converted to a system that disrupts processes in the cytoplasm and yield a transcriptome with CHX-like commonalties.

In addition to stresses or mutations that lead to the accumulation of photodynamic molecules, it is expected that plants deficient in the scavenging of basal levels of ^1^O_2_ production may show diagnostic ^1^O_2_ transcriptome signatures. For example, non-enzymatic lipid peroxidation was shown to activate stress signalling genes in the tocopherol-deficient mutant *vte2.* (Sattler et al., 2006). Inspection of the basal state of the *vte2* up-regulated transcriptome grown under moderate light conditions (120 µE m^-2^ s^-1^ under 16 h light/8 h dark cycle for 3 days) detects significant overlaps (33%, p-value = 7.4E-19) and (27%, p-value = 4.5E-36) with the transcriptomes of CHX and RB, respectively (**Supplemental Table S1A**). The TAI value was calculated to be TAI = 0.60 (**Supplemental Figure S4**), This suggests that a component of the cellular reaction brought about by the loss of scavenging capacity in the *vte2* mutant could be explained by its inability to detoxify ^1^O_2_ that initiates the attenuation of translation. Interestingly, it was observed that *Ch1* mutants lacking either tocopherol or zeaxanthin in the *Ch1vte2* and *Ch1npq1* mutants were more susceptible to cold/HL treatment than the *Ch1* mutant alone (Havaux et al., 2007). Hence, ^1^O_2_ confined to the chloroplast will lead to one set of transcriptomic responses while stresses that impact on chloroplast leakage may lead to another. The analyses presented here show the importance of localizing the source of photodynamic molecules in the cell that will contribute to their specific modes of action.

## Data availability statement

The original contributions presented in the study are publicly available. This data can be found here: NCBI, GSE205861.

## Author contributions

EK and RF designed the experiments. EK and AB executed the experiments. All authors participated in the interpretation and discussion of results. EK and RF wrote the manuscript. All authors contributed to the article and approved the submitted version.

## Funding

This work was supported by a grant from the Israel Science Foundation to RF, Grant number 2106/21.

## Acknowledgments

The authors would like to thank Tevi Mehlman, Dr. Hadas Keren-Shaul, Dr. Yosef Addadi and Dr. Bareket Dassa of the Weizmann Life Sciences Core Facility for excellent technical assistance. We would also like to thank Dr. Tomer Chen for invaluable advice and discussions in the course of this work.

## Conflict of interest

The authors declare that the research was conducted in the absence of any commercial or financial relationships that could be construed as a potential conflict of interest.

## Publisher’s note

All claims expressed in this article are solely those of the authors and do not necessarily represent those of their affiliated organizations, or those of the publisher, the editors and the reviewers. Any product that may be evaluated in this article, or claim that may be made by its manufacturer, is not guaranteed or endorsed by the publisher.
