# Supplementary material for: Plastid and cytoplasmic origins of 1O2-mediated transcriptomic responses
Source: Front Plant Sci. 2022 Nov 7;13:982610. doi: 10.3389/fpls.2022.982610 (PMC9676463; doi:10.3389/fpls.2022.982610)
Supplement: Supplementary file 1 [file DataSheet_1.pdf]

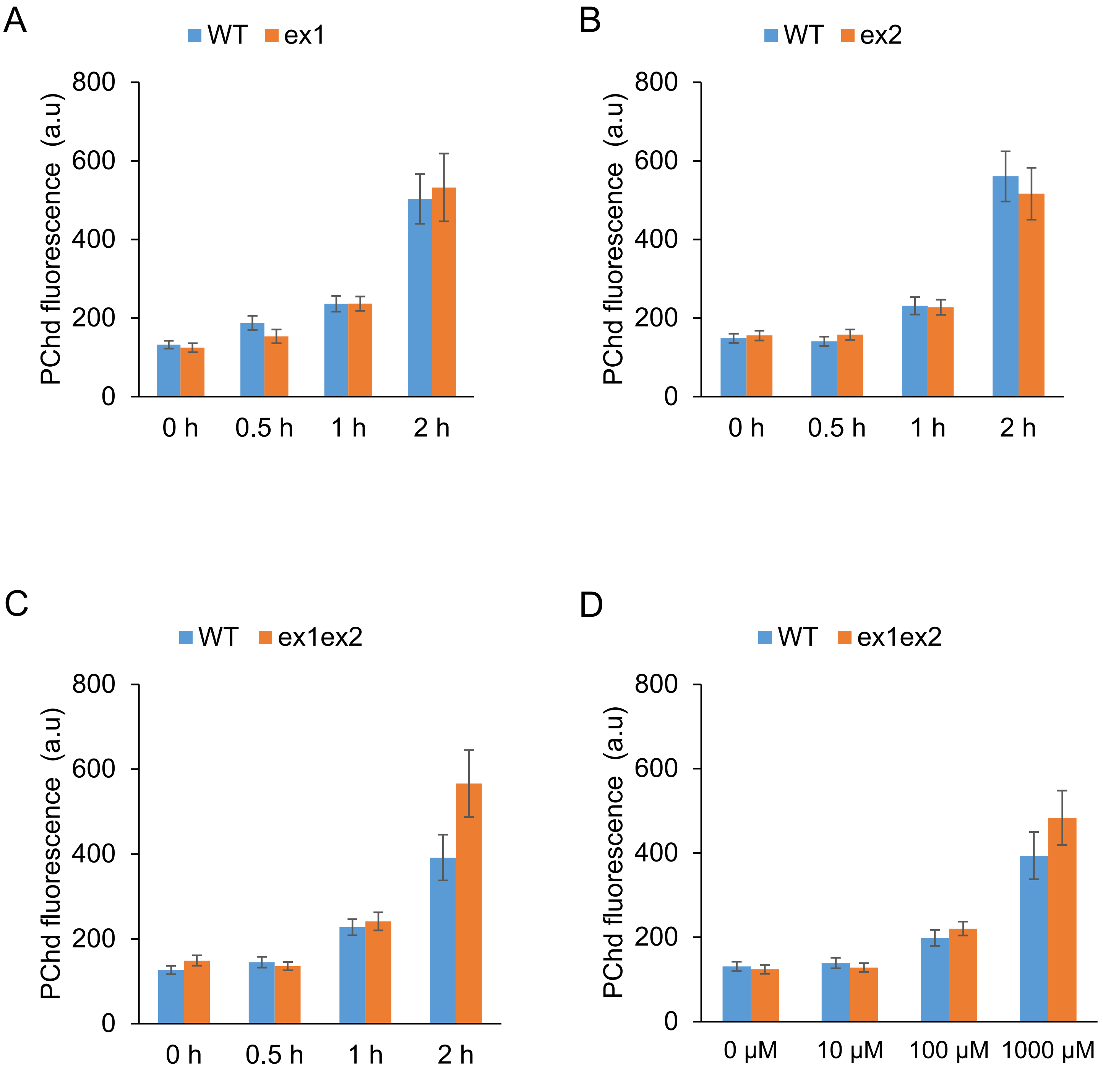

Supplemental Figure S1. Accumulation of Pchd is not affected by the executor pathway. **(A, B, C)** Protochlorophyllide (Pchd) accumulation in ALA-treated WT, *ex1*, *ex2* or *ex1ex2* seedlings under different time points. WT, *ex1*, *ex2* or *ex1ex2* plants were treated with 1 mM ALA (in DDW) and incubated for 0, 0.5, 1, 2 h in the dark before measurement.
(D) Protochlorophyllide (Pchd) accumulation ALA-treated WT and *ex1ex2* seedlings under different ALA concentrations. WT and *ex1ex2* plants were treated with 0, 10, 100, 1000 μM ALA (in DDW) for 2 h in the dark before measurement.
Fluorescence was measured using a fluorimeter (Ex/Em: 440/630 nm). The means and SE of 24 whole seedlings per time point are shown. No significant differences were found between the genotypes tested.

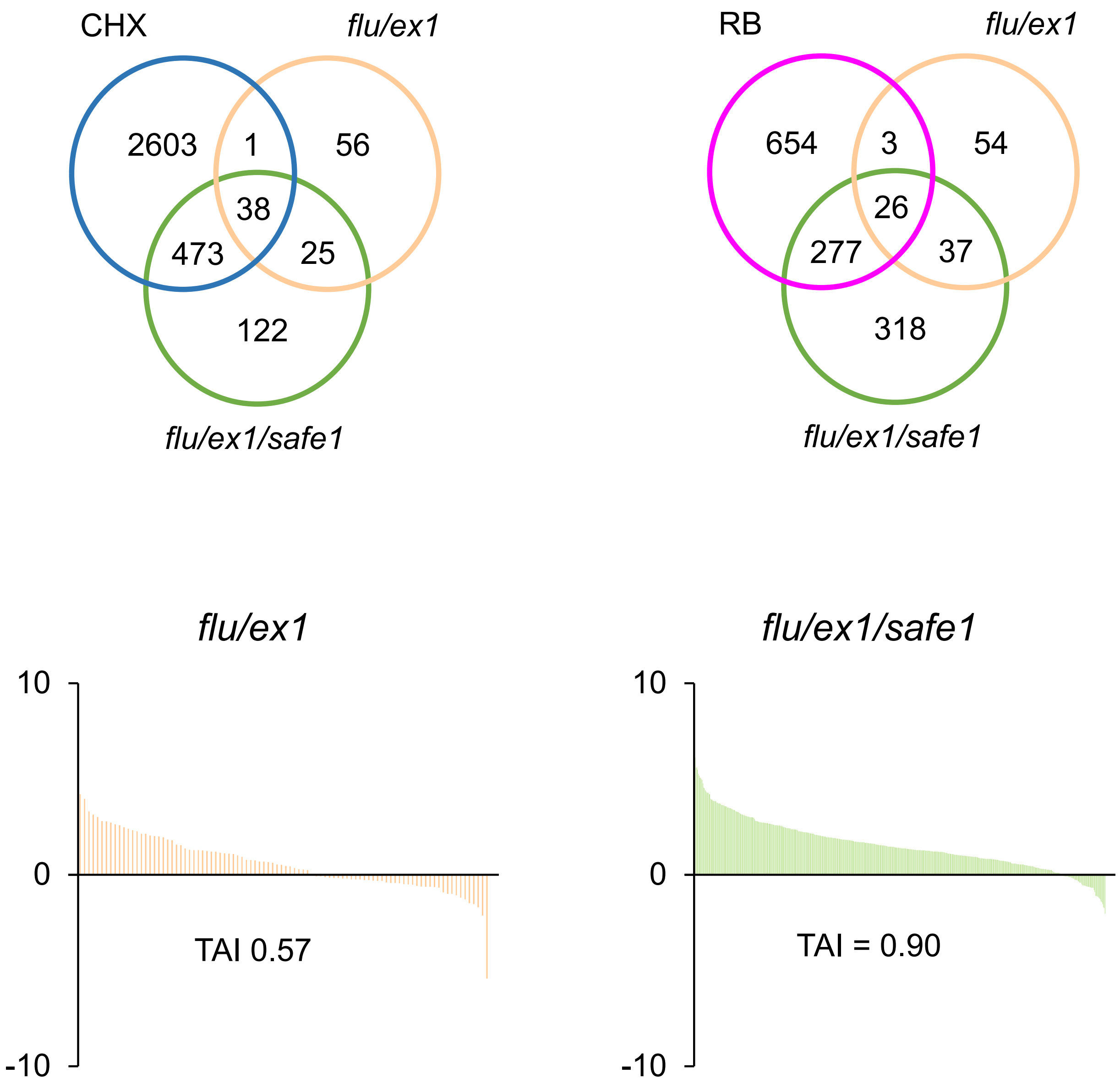

Supplemental Figure S2. Translational Attenuation Index (TAI) analysis of *flu/ex1* and *flu/ex1/safe1* transcriptomes after 4 h dark incubation followed by 30 min light (100 µE m^{-2} s^{-1}) exposure. The data here were obtained from public databases. (*flu/ex1, flu/ex1/safe1* - GSE131610; CHX – GSE111284; RB - GSE111285).

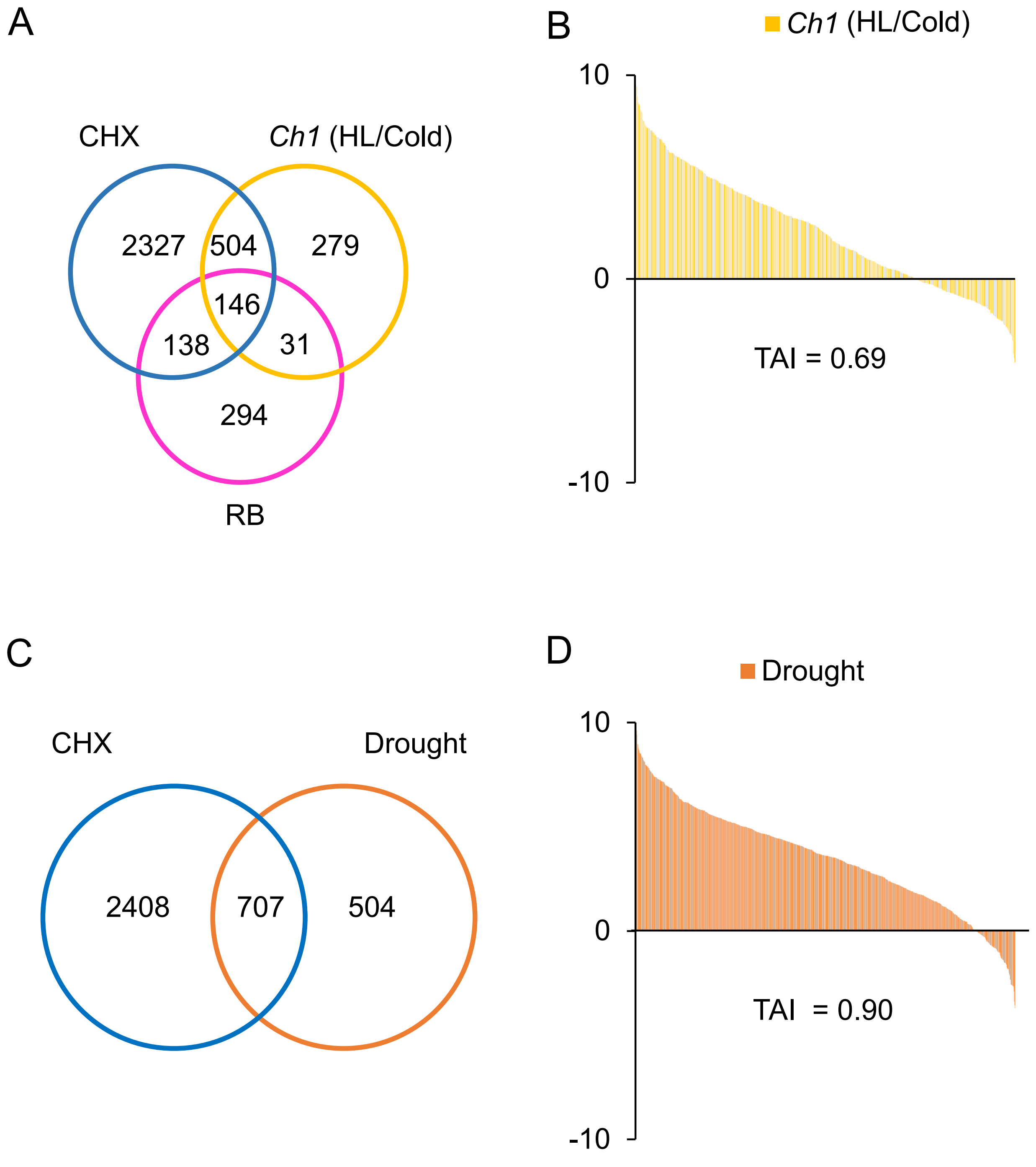

Supplemental Figure S3. Venn diagram and Translational Attenuation Index (TAI) analysis of *Ch1* and drought transcriptomes.

(A) Venn diagram overlaps of 2-fold induced genes from cycloheximide (CHX), RB, and *Ch1* (HL / Cold) mutant plants incubated under high light / cold stress (1200 µE m^{-2} s^{-1}, 10°C, 48 h, Ramel, 2013).

(B) Translational Attenuation Index analysis of *Ch1* (HL / Cold) induced transcripts.

(C) Venn diagram overlaps of 2-fold induced genes of Up-regulated of CHX and drought (1 h dry treatment on Whatman paper, Mizoguchi, 2010) transcriptome.

(D) Translational Attenuation Index analysis of drought transcripts.

The data from CHX, RB, *Ch1* (HL / Cold) and Drought were obtained from public databases. (CHX – GSE111284 ; RB - GSE111285; *Ch1* - Project CEA10-02_Light; Drought – ArrayExpress E-MEXP-2377).

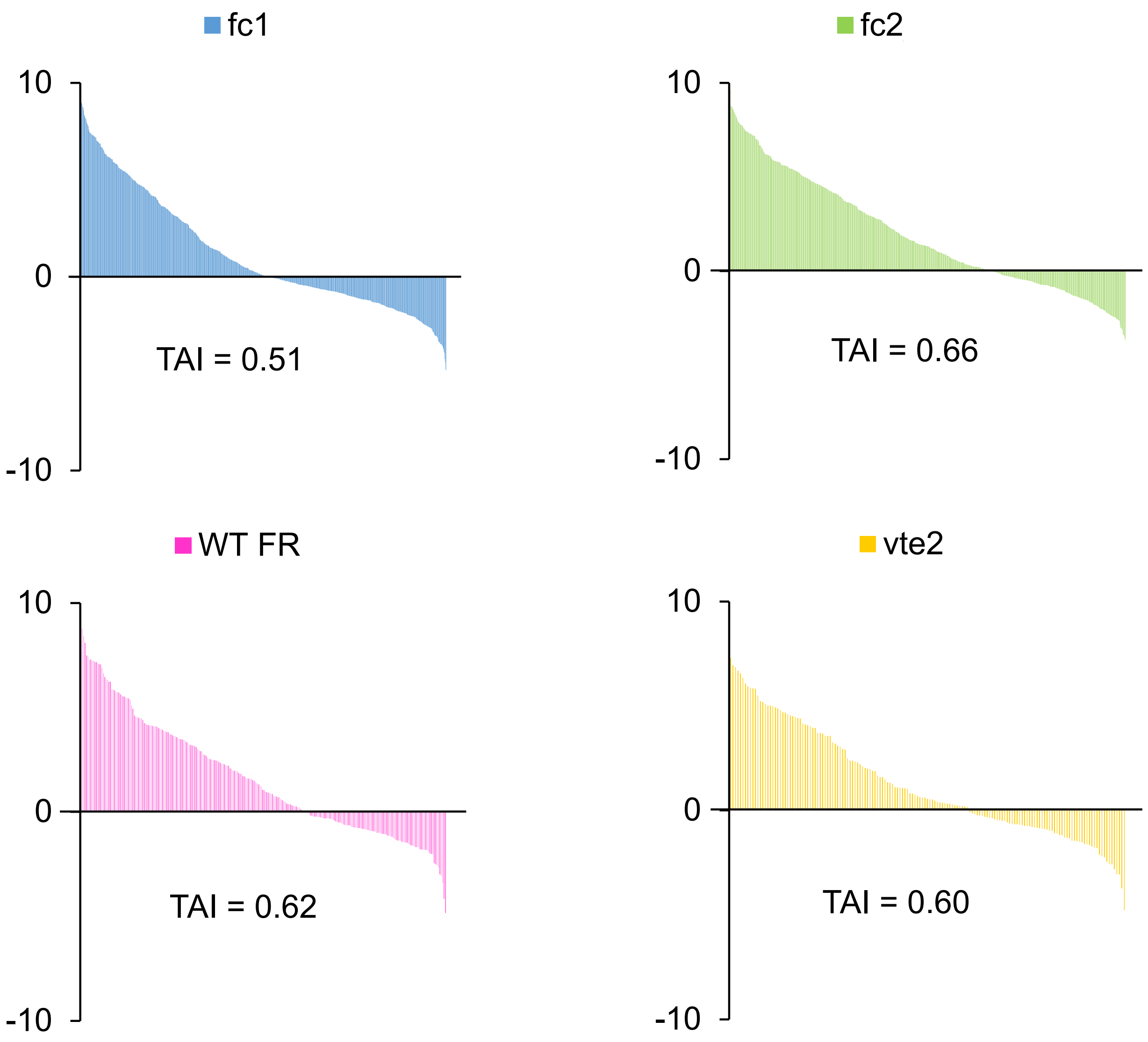

Supplemental Figure S4. Translational Attenuation Index (TAI) analysis of *fc1*, *fc2, vte2* mutants, Far-Red (FR) light treatment.

The data here were obtained from public databases. (*fc1*, *fc2* – GEO GSE71764; FR treatment – GSE6169; *vte2* - GSE4847, CHX – GSE111284,).

Overlap (%)	CHX	RB	*flu*	DCMU	BCC	*vte2*	*Ch1 (HL/Cold)*	MeJA	Far Red	*fc1*	*fc2*
CHX	100.0	67.7 (0)	49.2 (2.9E-70)	18.8 (8.9E-22)	31.4 (4.4E-23)	33.2 (7.4E-19)	46.6 (3.3E-118)	29.8 (6.2E-33)	28.1 (1.3E-25)	23.1 (2.9E-41)	29.0 (8.2E-71)
RB		100.0	39.9 (1.1E-106)	22.8 (4.4E-91)	24.2 (1.2E-41)	27.3 (4.5E-36)	29.1 (1.2E-119)	14.4 (3.9E-26)	25.3 (8.4E-68)	19.8 (7.6E-74)	25.2 (1.7E-132)
flu			100.0	28.5 (2.2E-72)	13.7 (5.4E-32)	10.2 (9.9E-15)	19.8 (5.7E-44)	7.8 (1.5E-10)	12.1 (5.0E-24)	20.4 (1.2E-26)	26.9 (3.7E-49)
DCMU				100.0	23.5 (3.6E-48)	10.2 (2.3E-8)	13.1 (5.6E-36)	6.6 (3.4E-36)	16.1 (8.6E-38)	21.6 (4.7E-65)	25.0 (2.3E-95)
BCC					100.0	16.1 (2.4E-30)	48.8 (1.1E-164)	8.9 (2.9E-12)	8.2 (1.5E-11)	25.6 (3.7E-37)	35.5 (3.5E-72)
vte2						100.0	25.4 (1.1E-41)	5.4 (5E-4)	17.6 (4.9E-28)	23.4 (2.1E-22)	27.3 (2.5E-32)
Ch1 (HL/Cold)							100.0	16.3 (1.9E-47)	13.8 (1.6E-32)	26.9 (9.9E-85)	30.4 (1E-115)
MeJA								100.0	4.5 (4.2E-5)	10.3 (1.1E-8)	15.2 (1E-24)
Far Red									100.0	19.9 (2.7E-34)	26.0 (3.9E-63)
fc1										100.0	46.8 (3.9E-63)
fc2											100.0

Supplemental Table S1A. Comparison of transcriptome overlap of up-regulated transcripts in different stress transcriptomes. Percentages represent overlap of the smaller gene set. P-values were obtained by using Fisher’s Exact Test of 2x2 contingency table for each individual comparison and are represented by the numbers in parentheses. Genes which showed greater than 2-fold increase in expression and a P value of $P<0.05$ in the various treatments were obtained from the raw datasets, processed using R and checked for overlaps. Databases; CHX - GSE111284; RB - GSE111285; *flu* - GSE111286; DCMU - GSE111287; BCC – GSE33963; *vte2* – GSE4847; *Ch1* – GSE34247 MeJA – AtGenExpress ME00337. Far Red – GSE6169, *fc1/fc2* – GSE71764.

Overlap (%)	CHX	RB	*flu*	DCMU	BCC	*vte2*	*Ch1 (HL/Cold)*	MeJA	Far Red	*fc1*	*fc2*
CHX	100.0	34.4 (2E-98)	31.5 (2.2E-98)	17.0 (9.2E-16)	37.5 (3E-17)	20.3 (3.7E-4)	34.0 (2.1E-70)	7.7 (0.49)	20.4 (4.6E-33)	14.2 (1.1E-17)	12.4 (8.2E-7)
RB		100.0	13.5 (1.7E-37)	11.5 (3.9E-25)	9.1 (5.2E-4)	6.8 (0.035)	8.7 (4.5E-14)	2.7 (0.48)	6.9 (1.1E-11)	6.8 (4.9E-6)	4.7 (1.9E-4)
flu			100.0	18.2 (3.9E-50)	19.3 (1.6E-9)	5.1 (0.18)	17.7 (3E-45)	4.1 (0.13)	9.0 (1.6E-16)	4.1 (0.14)	5.6 (5.6E-5)
DCMU				100.0	8.0 (5.9E-4)	3.4 (0.25)	6.9 (8E-12)	3.2 (0.098)	8.2 (2.1E-13)	10.7 (3.9E-14)	8.2 (1.3E-15)
BCC					100.0	0.0 (1)	46.6 (9E-51)	2.3 (0.14)	18.2 (3.6E-11)	14.8 (4.4E-6)	15.9 (5.2E-10)
vte2						100.0	6.8 (0.013)	0.0 (1)	11.9 (2.2E-4)	3.4 (0.71)	1.7 (1)
Ch1 (HL/Cold)							100.0	6.8 (2.1E-6)	19.0 (4.3E-58)	3.5 (0.69)	3.7 (0.0064)
MeJA								100.0	8.6 (2.3E-7)	10.4 (8.5E-7)	10.4 (1.5E-11)
Far Red									100.0	9.5 (1.4E-13)	6.1 (5.7E-11)
fc1										100.0	40.6 (1.5E-184)
fc2											100.0

Supplemental Table S1B. Comparison of transcriptome overlap of down-regulated transcripts in different stress transcriptomes. Percentages represent overlap of the smaller gene set. P-values were obtained by using Fisher's Exact Test of 2x2 contingency table for each individual comparison and are represented by the numbers in parentheses. Genes which showed greater than 2-fold decrease in expression and a P value of $P<0.05$ in the various treatments were obtained from the raw datasets, processed using R and checked for overlaps. Databases; CHX - GSE111284; RB - GSE111285; *flu* - GSE111286; DCMU - GSE111287; BCC – GSE33963; *vte2* – GSE4847; *Ch1* – GSE34247 MeJA – AtGenExpress ME00337. Far Red – GSE6169, *fc1/fc2* – GSE71764.

Overlap (%)	*Ch1* (Cold / HL)	*Ch1* LL 1 h	*Ch1* LL 2 h	*Ch1* LL 4 h
Ch1 (Cold / HL)	100.0	5.7 (1.2E-11)	5.7 (1.2E-9)	4.1 (5.6E-3)
Ch1 LL 1 h		100.0	60.4 (0)	56.3 (0)
Ch1 LL 2 h			100.0	67.8 (0)
Ch1 LL 4 h				100.0

Overlap (%)	*Ch1* (Cold / HL)	*Ch1* HL 1 h	*Ch1* HL 2 h	*Ch1* HL 4 h
Ch1 (Cold / HL)	100.0	8.9 (2.4E-19)	7.1 (9E-11)	6.6 (3.8E-5)
Ch1 HL 1 h		100.0	60.6 (0)	56.4 (0)
Ch1 HL 2 h			100.0	71 (0)
Ch1 HL 4 h				100.0

Supplemental Table S2. Comparison of transcriptome overlap of up-regulated transcripts of *Ch1* mutant plants subject to low light (30 µE m^{-2} s^{-1}) or high light (1000 µE m^{-2} s^{-1}) for 0, 1, 2, 4 h against *Ch1* transcriptomes obtained under cold / HL treatment (1200 µE m^{-2} s^{-1}, 10°C, 48 h, see Ramel, 2013). Percentages represent overlap of the smaller gene set. P-values were obtained by using Fisher's Exact Test of 2x2 contingency table for each individual comparison and are represented by the numbers in parentheses. Genes which showed greater than 2-fold increase in expression and a P value of $P<0.05$ in the various treatments were obtained from the raw datasets, processed using R and checked for overlaps. The transcriptomes of *Ch1* (Ramel), *Ch1* LL/HL were obtained from the relevant online databases. *Ch1* (Cold / HL) - CEA10-02_Light, *Ch1* LL/HL – GSE205861.

Overlap (%)	DCMU	*Ch1* LL 1 h	*Ch1* LL 2 h	*Ch1* LL 4 h
DCMU	100.0	31.6 (3.3E-119)	21.4 (3E-74)	16.6 (2.9E-62)
Ch1 LL 1 h		100.0	60.4 (0)	56.3 (0)
Ch1 LL 2 h			100.0	67.8 (0)
Ch1 LL 4 h				100.0

Overlap (%)	DCMU	*Ch1* HL 1 h	*Ch1* HL 2 h	*Ch1* HL 4 h
DCMU	100.0	26.8 (1.3E-161)	25.6 (4.1E-140)	23.2 (3.8E-93)
Ch1 HL 1 h		100.0	60.6 (0)	56.4 (0)
Ch1 HL 2 h			100.0	71 (0)
Ch1 HL 4 h				100.0

Supplemental Table S3. Comparison of transcriptome overlap of up-regulated transcripts of *Ch1* mutant plants subject to low light (30 μE m^{-2} s^{-1}) or high light (1000 μE m^{-2} s^{-1}) for 0, 1, 2, 4 h against DCMU transcriptomes obtained under HL treatment (1000 μE m^{-2} s^{-1}, 2 h, see Koh, 2021). Percentages represent overlap of the smaller gene set. P-values were obtained by using Fisher's Exact Test of 2x2 contingency table for each individual comparison and are represented by the numbers in parentheses. Genes which showed greater than 2-fold increase in expression and a P value of $P<0.05$ in the various treatments were obtained from the raw datasets, processed using R and checked for overlaps. The transcriptomes of DCMU, *Ch1* LL/HL were obtained from the relevant online databases. DCMU - GSE111287, *Ch1* LL/HL – GSE205861

Overlap (%)	BCC	*Ch1* LL 1 h	*Ch1* LL 2 h	*Ch1* LL 4 h
BCC	100.0	6.8 (2E-8)	5.8 (2E-5)	4.8 (9.2E-3)
Ch1 LL 1 h		100.0	60.4 (0)	56.3 (0)
Ch1 LL 2 h			100.0	67.8 (0)
Ch1 LL 4 h				100.0

Overlap (%)	BCC	*Ch1* HL 1 h	*Ch1* HL 2 h	*Ch1* HL 4 h
BCC	100.0	15 (5.8E-25)	11.3 (6E-14)	10.6 (9.4E-9)
Ch1 HL 1 h		100.0	60.6 (0)	56.4 (0)
Ch1 HL 2 h			100.0	71 (0)
Ch1 HL 4 h				100.0

Supplemental Table S4. Comparison of transcriptome overlap of up-regulated transcripts of *Ch1* mutant plants subject to low light (30 µE m^{-2} s^{-1}) or high light (1000 µE m^{-2} s^{-1}) for 0, 1, 2, 4 h against the BCC transcriptome (Ramel, 2012b, D'Alessandro, 2018). Percentages represent overlap of the smaller gene set. P-values were obtained by using Fisher's Exact Test of 2x2 contingency table for each individual comparison and are represented by the numbers in parentheses. Genes which showed greater than 2-fold increase in expression and a P value of P<0.05 in the various treatments were obtained from the raw datasets, processed using R and checked for overlaps. The transcriptomes of BCC, *Ch1* LL/HL were obtained from the relevant online databases. BCC - CEA10-03_Cyclocitral, *Ch1* LL/HL – GSE205861
